# A Nondestructive Method to Quantify Polymer Graft Density on Nanoparticles Using Optical Extinction Spectra

**DOI:** 10.1021/acsnano.6c11390

**Published:** 2026-09-29

**Authors:** Masoud Abdi, Irene Andreu, Ryan Poling-Skutvik

**Affiliations:** † Department of Chemical, Biomolecular, and Materials Engineering, 4260 University of Rhode Island, Kingston, Rhode Island 02881, United States; ‡ Department of Physics, University of Rhode Island, Kingston, Rhode Island 02881, United States

**Keywords:** localized surface plasmon resonance, polymer-grafted nanoparticles, grafting density, polymer brushes, refractive index sensitivity, plasmonic nanoparticles

## Abstract

Grafting density is the key parameter governing colloidal stability and structure for polymer-grafted nanoparticles (PGNPs). Existing methods to quantify grafting density either destroy the sample, require prior knowledge of polymer molecular weight *M*_n_, or rely on complex instrumentation. Here, we present a nondestructive optical approach that determines grafting density directly from shifts in the localized surface plasmon resonance peak caused by the grafted polymer changing the local effective refractive index. To interpret this shift, the grafted polymer chains are approximated as cylindrical segments passing through differential spherical shells to obtain radial profiles for the polymer volume fraction and refractive index across the polymer corona. The local refractive-index profile is weighted by the plasmonic near-field decay to calculate the effective refractive index sensed by the nanoparticle. The grafting density is finally determined by iteratively adjusting the effective refractive index until the calculated refractive-index sensitivity of the polymer-grafted nanoparticle matches that of the gold core. The grafting densities obtained from this optical method quantitatively agree with established experimental benchmarks across different polymer *M*_n_, grafting densities, nanoparticle sizes, and solvents. Our approach uses common laboratory equipment, eliminates the need for prior knowledge of polymer *M*_n_, and preserves the sample for further experiments, bypassing challenges provided by existing characterization approaches. Together, these advantages provide a framework for predicting and understanding the physical and chemical properties of PGNPs for specific applications.

## Graphical abstract

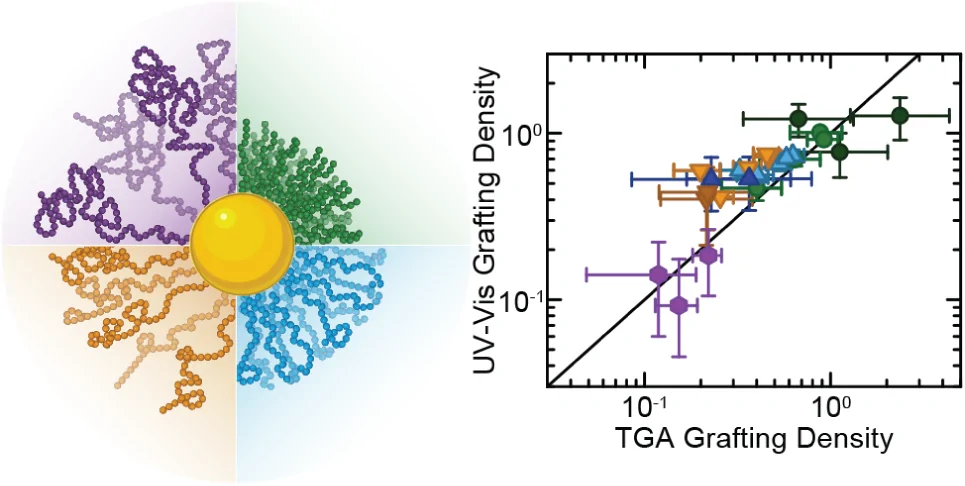

## 1 Introduction

Metallic nanoparticles exhibit localized surface plasmon resonance (LSPR), which is a phenomenon that arises when an incident electromagnetic field couples to the free conduction electrons in the nanoparticle.^1^ The LSPR sensitivity to the local dielectric environment has enabled the use of plasmonic nanoparticles in various applications including biosensing,^2^ drug delivery,^3^ photothermal therapy,^4^ solar cells,^5^ catalysis,^6^ photoacoustic imaging,^7^ and surface-enhanced Raman spectroscopy.^8,9^ This sensitivity has enabled impressive success in the quantitative monitoring of molecular adsorption and binding on AuNP surfaces through LSPR peak shifts.^10–13^ In many applications, however, nanoparticles aggregate under attractive interactions introduced by van der Waals forces or electrostatics, for example, quenching the LSPR response and reducing their performance.^14,15^ To promote stability, nanoparticles are commonly functionalized with polymer chains to form a tunable stabilizing interfacial layer.^16–18^ The result is a polymer-grafted nanoparticle (PGNP) whose stability is largely governed by the properties of the tethered chains, especially the grafting density σ.^19,20^ Consequently, measuring σ is crucial for characterizing PGNPs for both fundamental investigations and practical applications.

Thermogravimetric analysis (TGA) is the most common technique to measure grafting density, where σ is estimated from polymer mass loss during thermal decomposition.^21–25^ Despite its ubiquity, TGA suffers from significant drawbacks. It is a fully destructive technique that requires ∼1 mg of sample, which can be prohibitive when PGNP synthesis yields are low or costly. Additionally, converting mass loss into σ requires prior knowledge of polymer molecular weight *M*_n_, often necessitating additional polymer characterization using techniques such as gel permeation chromatography (GPC), which increases experimental cost and complexity.^26–28^ Other destructive methods have similar limitations. Electrochemical reductive desorption estimates σ by cleaving thiol–surface bonds and measuring the desorption charge, but competing reactions can overestimate surface coverage and the required voltammetry instrumentation may not be routinely available in many nanomaterials laboratories.^29^ The silver ion probe method quantifies bound ions by mass spectrometry, but incomplete or nonselective ion recovery during the multistep process can propagate substantial errors.^30^ Likewise, total organic carbon (TOC) analysis is destructive because the entire sample is acidified and oxidized to quantify carbon content. In this approach, residual free polymer or contaminants left after purification can be misidentified as grafted polymer, leading to overestimation of grafting densities.^31^

By contrast, nondestructive approaches aim to preserve the sample during characterization but they are often limited by relying on indirect measurements, model assumptions, and instrumental complexity. Nuclear magnetic resonance (NMR) spectroscopy estimates σ by integrating characteristic proton signals from the grafted polymer relative to a standard.^32^ However, the NMR method is limited by the difficulty of accurately quantifying polymer-derived protons relative to other signals in the sample, particularly when peak overlap, signal broadening, or baseline uncertainty obscure the integrated peak areas.^33,34^ When *M*_n_ is unknown, the NMR method can become destructive because grafted chains may need to be cleaved from the nanoparticle surface for independent *M*_n_ characterization.^33^ Analytical ultracentrifugation estimates σ indirectly from sedimentation behavior, which reflects both particle mass and hydrodynamic diameter. Accurate analysis requires prior knowledge of *M*_n_, a monodisperse core diameter, and reliable polymer-shell thickness, since uncertainty in these parameters can propagate into the calculated grafting density.^31^ Spectroscopic ellipsometry can estimate σ from the dry polymer-layer thickness, polymer density, and *M*_n_ only when particles form a uniform layer on a flat substrate. The accuracy of this approach is limited by the optical model used to extract thickness.^35^ Infrared spectroscopy estimates σ indirectly by correlating characteristic polymer vibrational bands with calibrated polymer loading curves, but peak overlap, baseline correction, and background signals from the metallic nanoparticles can affect accuracy.^36^ Atomic force microscopy (AFM) measures σ by fitting force–distance curves, but strong tip–polymer interactions can deform polymer brushes. Its measurements are also sensitive to tip shape and surface heterogeneity, and it requires expertise in both instrument operation and data analysis.^37^ Finally, X-ray photoelectron spectroscopy (XPS)^38,39^ and small-angle neutron scattering (SANS)^40^ offer indirect assessments of σ, but both require highly specialized instrumentation, advanced data processing, and simplifying analytical assumptions.

Due to these technological limitations, a widely deployable, nondestructive method for directly quantifying σ still remains to be developed. Here, we introduce an approach for quantifying σ from LSPR shifts using widely available UV–visible extinction spectroscopy (UV–vis). The method first calculates the effective local refractive index generated by the polymer corona using a plasmonic near-field model. An analytical assumption for the polymer brush conformation is then used to convert this optical response into a quantitative measurement of σ. Our analytical approach is tested across multiple polymer *M*_n_, grafting densities, particle sizes, and solvent choices with excellent quantitative agreement with TGA benchmarks. Unlike current methods, this approach is straightforward, nondestructive, and widely accessible. Finally, we provide an open-source application that directly calculates σ from UV–vis measurements to facilitate broad implementation of this method within the plasmonic nanoparticle community.

## 2 Results and Discussion

We prepare PGNPs with AuNP cores with sizes *R*_NP_ = 7 ± 1 and 31 ± 4 nm, and characterize the PGNP structure with TEM, as shown in Figure 1. TEM images demonstrate that all PGNPs present the same core radius with no noticeable aggregation following functionalization. The increasing edge-to-edge distances between functionalized particles with higher *M*_n_ indicate that the polymer corona thickness *h* increases with *M*_n_, consistent with theoretical predictions and our earlier work.^41,42^

**Figure 1:**
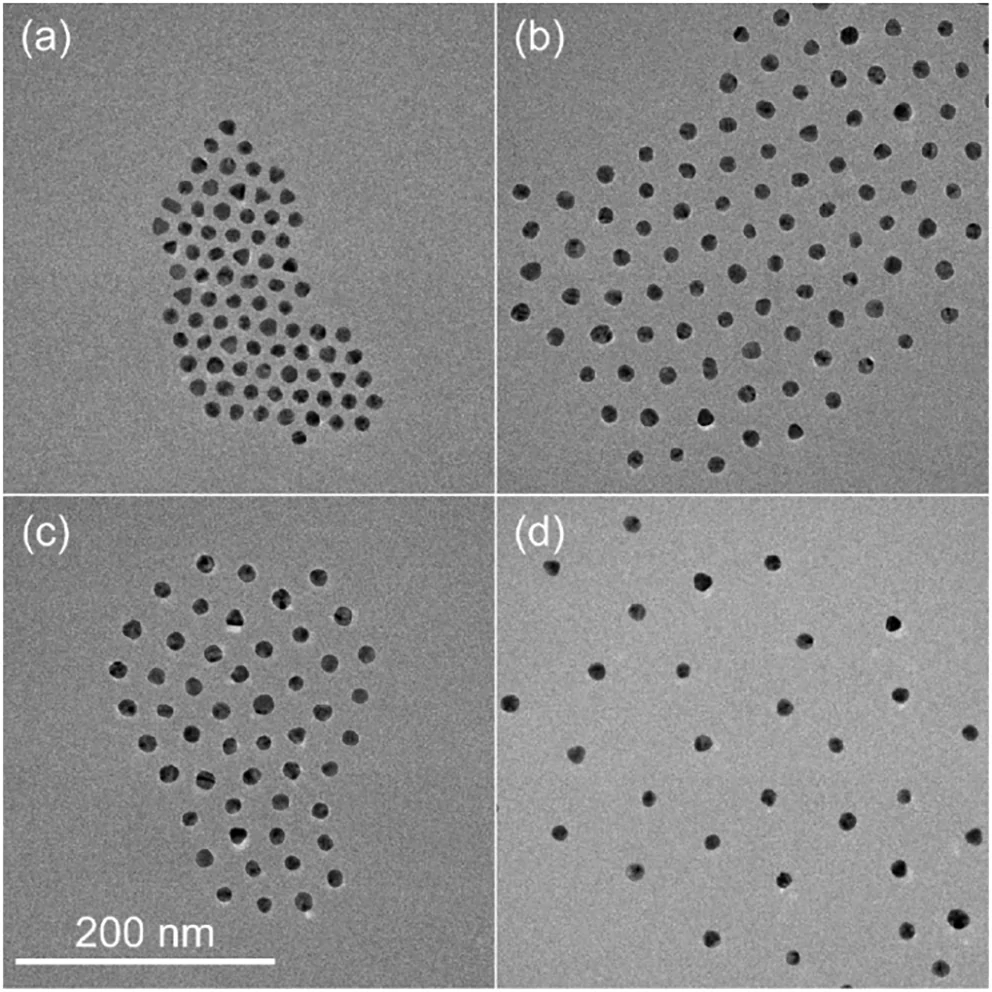
TEM images of polystyrene-functionalized AuNPs with *R*_NP_ = 7 nm with increasing polymer molecular weights of (a) 5.8 kDa, (b) 27 kDa, (c) 61 kDa, and (d) 259 kDa.

After confirming successful grafting of PS-SH to the AuNPs through TEM images, we quantify the grafting density σ of polymer chains on the PGNP surface using TGA. The PGNPs show significant mass loss in the range 300–450 °C corresponding to the thermal decomposition of the grafted polystyrene (Figure 2a and Supporting Information).^43–45^ TGA results show a significant increase in weight loss with increasing *M*_n_, for example increasing from 18% for PS5.8 to 56% for PS259 on AuNPs with *R*_NP_ = 7 nm, indicating more polymer mass on the AuNPs surface.

**Figure 2:**
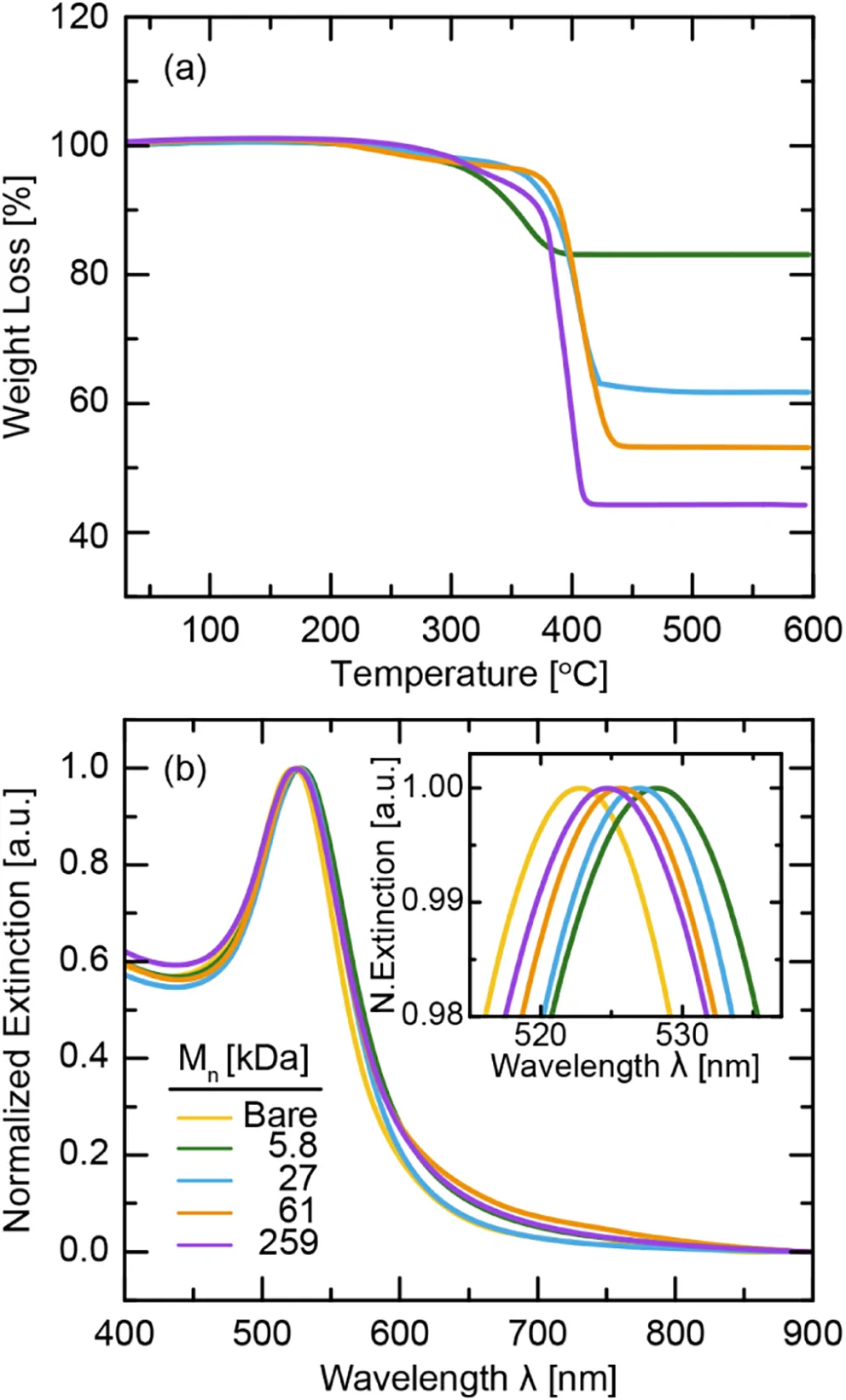
(a) Weight loss of PGNPs with *R*_NP_ = 7 nm functionalized with various *M*_n_ at similar concentrations as a function of temperature under a heating rate of 5 °C min^–1^. (b) Normalized UV–vis extinction spectra for PGNPs with *R*_NP_ = 7 nm as a function of wavelength λ, for bare AuNPs in a water-glycerol solution (*n* = 1.4116) and PGNPs with different *M*_n_ in THF (*n* = 1.4116).

Additionally, this PS functionalization promotes stability in organic solvents^42,46^ which we characterize through the PGNP optical response. All PGNPs exhibit a narrow, symmetric LSPR peak (Figure 2b and Supporting Information), indicating that PGNPs are stable in THF. For all PGNPs, we observe red shifts in the LSPR peak position relative to that of bare AuNPs. This red shift is expected because PS has a higher refractive index than the surrounding solvent and therefore increases the local dielectric environment near the AuNP surface.

TGA and UV–vis results, however, show opposite trends with polymer *M*_n_. As *M*_n_ increases, the total grafted polymer mass measured by TGA increases, whereas the LSPR red shift measured by UV–vis decreases. For example, we find that the low *M*_n_ sample (5.8 kDa) exhibits the largest red shift, from 523 to 529 nm, but has the lowest weight loss of 18%. This contrast in trends between TGA and UV indicates that the LSPR response is not governed by the total polymer mass; instead, we hypothesize that the peak shift must be controlled by σ, which quantifies the density of polymer near the AuNP surface and thereby the local dielectric environment.

If the observed red shift results from the polymer corona increasing the local refractive index within the LSPR near field, the effect should be described by Mie theory,^47^ which solves the differential equations underlying the electromagnetic response.^48,49^ We calculate the extinction spectra for AuNPs using the MiePlot software^50,51^ with the McPeak dielectric function for gold.^52^ These calculations show excellent agreement with the measured LSPR peak of bare AuNPs of both sizes suspended in water–glycerol mixtures, quantitatively reproducing the peak position and qualitatively matching the experimental peak shape (Supporting Information). Following this success, we use Mie calculations to predict the LSPR peak position of bare AuNPs of both sizes across the refractive-index range of organic solutions of THF and toluene and compare those predictions to measurements for PGNPs suspended in the same solutions (Figure 3 and Supporting Information). Indeed, Mie theory calculations for bare AuNPs show that peak shift Δλ_max_ increases linearly with refractive-index. For AuNPs with *R*_NP_ = 7 nm, the total peak shift is 5.9 nm as refractive-index increases from *n* = 1.4116 (THF) to *n* = 1.5027 (toluene), as shown in Figure 3b. The AuNPs with *R*_NP_ = 31 nm exhibit a corresponding total peak shift of 11.5 nm over the same refractive-index range (Supporting Information). We find that all PGNP samples show similar linear increases in Δλ_max_ over the same range in solvent refractive index *n*_solv_, but with different magnitudes for each *M*_n_. For example, PGNPs with *R*_NP_ = 7 nm with high and low *M*_n_ exhibit peak shift magnitudes of 5.5 and 3.8 nm, respectively, which is significantly smaller than the predicted shift of 5.9 nm for bare AuNPs. In Mie theory, the LSPR response depends only on the particle size, geometry, and the dielectric environment, and from TEM, we confirm that there is no change in particle size or shape during functionalization. Therefore, the observed differences in the magnitude of the peak shifts between bare AuNPs and PGNPs must result from differences in the local dielectric environment caused by the grafted polymer corona within the plasmonic near-field decay.^1,53^

**Figure 3:**
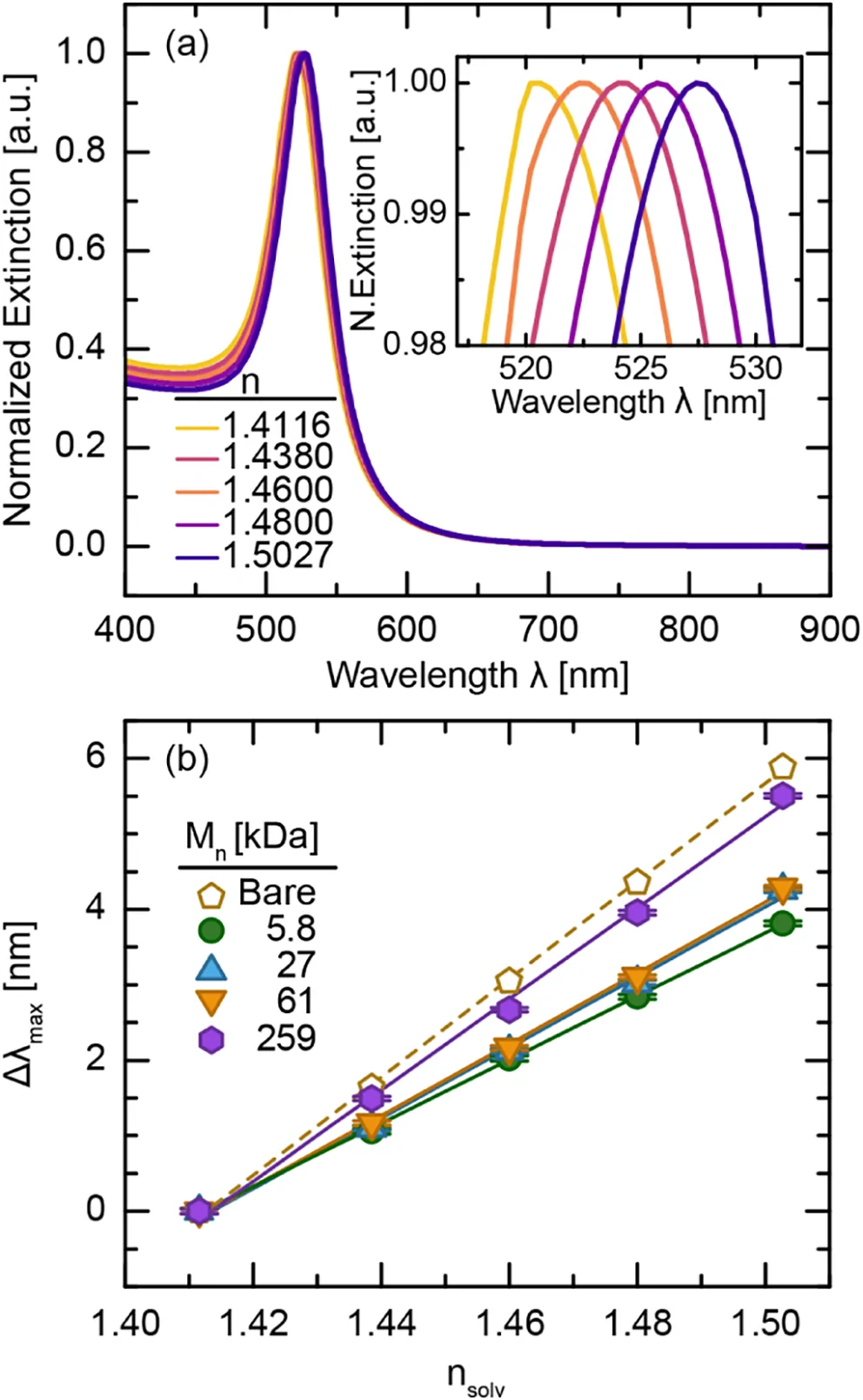
(a) Predicted UV–vis extinction spectra from Mie theory as a function of wavelength λ for bare AuNPs with *R*_NP_ = 7 nm in solutions of varying refractive indices. (b) Peak shift Δλ_max_ of PGNPs with *R*_NP_ = 7 nm (closed symbols) from pure THF and Mie theory predictions for bare AuNPs (open symbols) as a function of the refractive index of THF–toluene mixtures. Solid lines show linear fits to the experimental data, and dashed lines show linear fits to the calculated data.

Because the plasmonic field decays exponentially with distance from the AuNP surface, monomers near the particle surface contribute more strongly to the resulting LSPR shift than those farther from the surface. Earlier studies^54–56^ identified an empirical relationship between LSPR peak position and the change in local index of refraction Δ*n* according to

1 $$\Delta \lambda_{\max}=m\Delta n\left[1-\exp\left(-2d/L\right)\right]$$

where *m* is the refractive index sensitivity (RIS), *d* is the thickness of a bound analyte layer, and *L* is the electromagnetic field decay length that depends on particle size and geometry.^56–58^ If *d* ≫ *L* (e.g., when a particle is transferred to a different solvent so that *d* → ∞), the peak shift becomes a linear function of the refractive index change Δλ_max_ = *m* Δ*n*. Our Mie theory calculations confirm this relationship with *m* = 64.5 ± 0.6 nm per refractive index unit (RIU) for our bare AuNPs with *R*_NP_ = 7 nm over the range of *n* spanned by mixtures of THF and toluene (Figure 3b). For the larger AuNPs, the corresponding Mie-predicted RIS is *m* = 125.5 ± 1.5 nm/RIU over the same range of *n* (Supporting Information). We cannot experimentally measure the peak shift of bare particles over the same range of *n* because the particles are unstable in organic solvents without PS functionalization, but we do experimentally confirm this linear relationship in aqueous solutions (Supporting Information). Therefore, we use the Mie-predicted RIS *m* for each AuNP core size as a reference to interpret the different shift magnitudes of PGNPs. We expect that differences in the RIS *m* with polymer *M*_n_ arise from changes in σ.

To relate peak shifts to σ, we develop a physical model schematically illustrated in Figure 4. In this model, we relate the macromolecular conformation of the grafted polymer chains, controlled by σ, to the radially dependent polymer volume fraction φ­(*z*) surrounding the particle, where *z* is the radial distance from the AuNP surface. We then use φ­(*z*) to calculate the distance-dependent refractive index *n*(*z*), which can be appropriately weighted to result in an effective refractive index *n*_eff_ controlling the peak shift Δ λ_max_. When implementing this model, we iterate over different σ so that the RIS *m* as a function of *n*_eff_ for the PGNPs converges to that of the corresponding bare AuNP core size. With this approach, we quantitatively extract the PGNP σ from peak shifts in solvent mixtures. We now provide more specifics on this approach.

**Figure 4:**
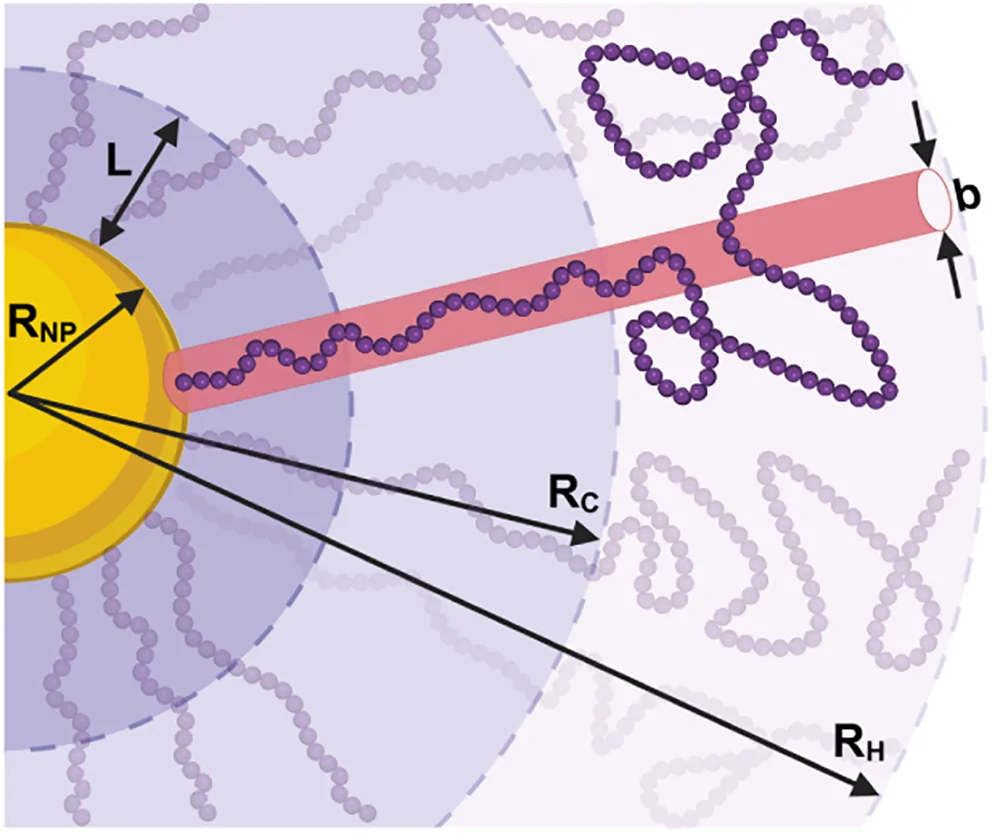
Schematic illustration of the important length scales of the PGNP model for predicting optical shifts: nanoparticle core radius *R*_NP_, decay length *L*, cross over radius *R*_C_, hydrodynamic radius *R*_H_, and effective cross-sectional diameter of a polymer chain *b*.

First, we build an analytical estimate of polymer density in the grafted corona as a function of σ. Near surfaces, grafted polymers^41,59^ exhibit extended conformations^60–62^ in which the polymer backbone orients normal to the surface. A concentrated polymer brush (CPB) regime exists at sufficiently high σ, in which the chain conformation can be approximated as a cylinder normal to the surface with a diameter *b*.^63^ Farther from the surface, the polymer density decreases and the chains enter a semidilute polymer brush (SDPB) regime with a more coiled conformation.^40,64,65^ The crossover radius between these two regimes is given by

2 $$R_{c}=R_{\mathrm{NP}}\sigma^{*1/2}\nu^{*-1}$$

where ν* = (4π)^1/2^ ν is a normalized excluded volume parameter with ν ≈ 0.588 for a good solvent condition^66^ and σ* = 4*π b* ^2^ σ normalizes the grafting density by the cross-sectional diameter *b* of the grafted chain. Typically, this diameter is assumed to be equivalent to the Kuhn length, but to achieve quantitative agreement in our model, we use an experimentally measured value *b* = 0.95 nm for polystyrene.^67^ For simplicity, we assume that the polymer chains exist primarily within the CPB regime, allowing us to approximate the polymer volume fraction as a function of radial distance *z* according to the ratio of the differential volume pervaded by the grafted chains to the differential volume of a spherical shell. Mathematically, this ratio is given by

3 $$\varphi \left(z\right)=\{\begin{array}{ll} \frac{\sigma \pi b^{2}R_{\mathrm{NP}}^{2}}{4{\left(R_{\mathrm{NP}}+z\right)}^{2}}, & 0<z\leq h\\ 0, & z>h \end{array}$$

where *h* is the thickness of the polymer corona calculated from the difference between the hydrodynamic radius *R*_*H*_ of the PGNP and *R*_NP_ in water. We validate this assumption and identify its limiting conditions further in the text. Using this approach, the φ­(*z*) profiles exhibit a dense polymer region near the particle surface that monotonically decays toward zero at large *z*, consistent with literature findings.^68^

Second, we convert φ­(*z*) into a spatially varying refractive index profile *n*(*z*) through the Lorentz–Lorenz relationship,^69^ which considers the dielectric effects of both polymer and solvent, given by

4 $$\frac{n{\left(z\right)}^{2}-1}{n{\left(z\right)}^{2}+2}=\varphi \left(z\right)\frac{n_{\mathrm{poly}}^{2}-1}{n_{\mathrm{poly}}^{2}+2}+\left[1-\varphi \left(z\right)\right]\frac{n_{\mathrm{solv}}^{2}-1}{n_{\mathrm{solv}}^{2}+2}$$

where *n*_poly_ = 1.5993 and *n*_solv_ are the refractive indices of polystyrene^70,71^ and solvent, respectively. The resulting profiles are shown in Figure 5b. Because changes in index farther from the particle surface cause weaker peak shifts (eq 1), we follow an approach from literature^55^ to calculate an effective index *n*_eff_ controlling the LSPR peak position by integrating *n*(*z*) with respect to a normalized weighting function $w\left(z\right)=\frac{2}{L}\exp\left(-2z/L\right)$ , according to

5 $$n_{\mathrm{eff}}=\frac{2}{L}\int_{0}^{\infty }n(z)\;\exp\left(-2z/L\right)dz$$

where *L* = 6.5 ± 0.1 nm and *L* = 31.6 ± 0.4 nm for the small and large AuNPs, respectively, determined from Mie theory calculations (Supporting Information). These values are consistent with those reported in literature.^13,54^ With this weighting, grafted chains near the particle surface contribute more significantly to peak shifts than those farther from the surface.

**Figure 5:**
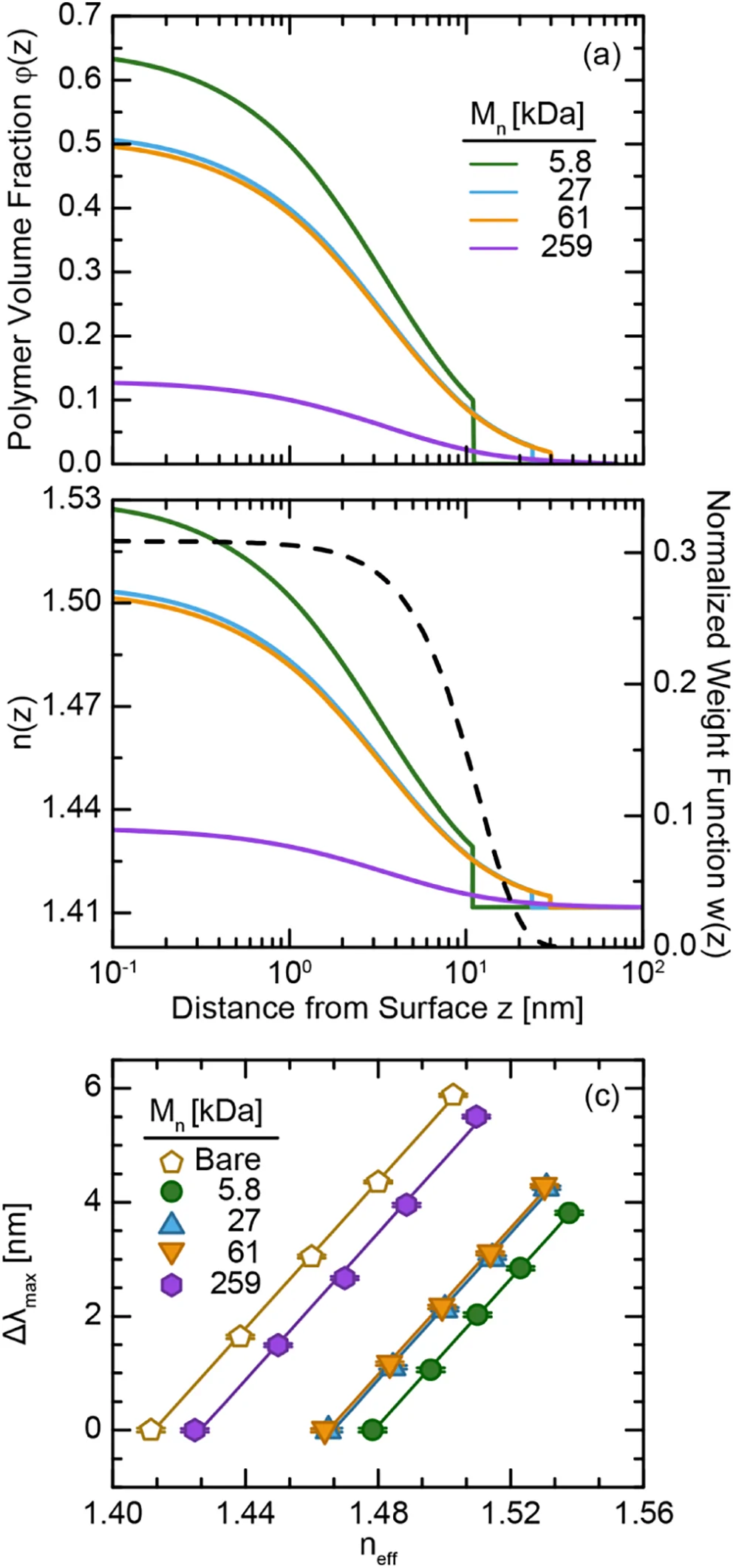
(a) Polymer volume fraction φ­(*z*) as a function of distance *z* from the nanoparticle surface for PGNPs with *R*_NP_ = 7 nm with various *M*_n_. (b) Corresponding local refractive index *n*(*z*) as a function of distance, with the dashed line indicating the normalized near-field electromagnetic decay weighting function *w*(*z*). (c) Mie theory predictions for bare AuNPs with *R*_NP_ = 7 nm (open symbols) as a function of the refractive index of THF–toluene mixtures, and peak shift Δλ_max_ as a function of *n*_eff_ for PGNPs with *R*_NP_ = 7 nm (closed symbols) with different *M*_n_. The solid and dashed lines indicate linear fits showing a constant slope with *m* = 64.5 ± 0.6.

Finally, we use these estimates of φ­(*z*) and *n*(*z*) to quantify σ through iterative calculations until the RIS of the PGNPs as a function of *n*_eff_ agrees with that of the corresponding bare AuNP core sizes. For PGNPs with *R*_NP_ = 7 nm, we find unique numerical convergence to *m* = 64.5 ± 0.6 nm/RIU for all *M*_n_, resulting in identical slopes of Δλ_max_ as a function *n*_eff_ at the converged graft density (Figure 5c). The PGNPs with *R*_NP_ = 31 nm similarly converge to the corresponding bare-particle RIS of *m* = 125.5 ± 1.5 nm/RIU (Supporting Information). For each solvent mixture, *n*_eff_ varies with *M*_n_ due to changes in σ, with higher σ shifting *n*_eff_ toward the polymer refractive index and lower σ yielding values closer to those of the pure solvent. For example, we find that PGNPs with *R*_NP_ = 7 nm with 5.8 kDa grafted polymer experience a higher *n*_eff_ than PGNPs with 259 kDa polymer because of a higher grafting density, consistent with the TGA results (Figure 2). This observation is consistent with our expectations that larger polymer chains experience more significant steric hindrances during “grafting-to” synthesis, resulting in lower σ.^72–74^

So far, we have established an analytical framework to quantitatively relate shifts in LSPR peaks to σ. We verify the accuracy of this approach by comparing the σ values extracted using this UV–vis method with those determined independently using TGA analysis, as shown in Figure 6. We find excellent agreement over a wide range of functionalization conditions for both AuNP core sizes. Specifically, the UV–vis and TGA estimates correlate with each other across an order of magnitude in σ and two orders in *M*_n_. This high degree of correlation between the methods indicates that the optical method accurately quantifies σ for plasmonic nanoparticles.

**Figure 6:**
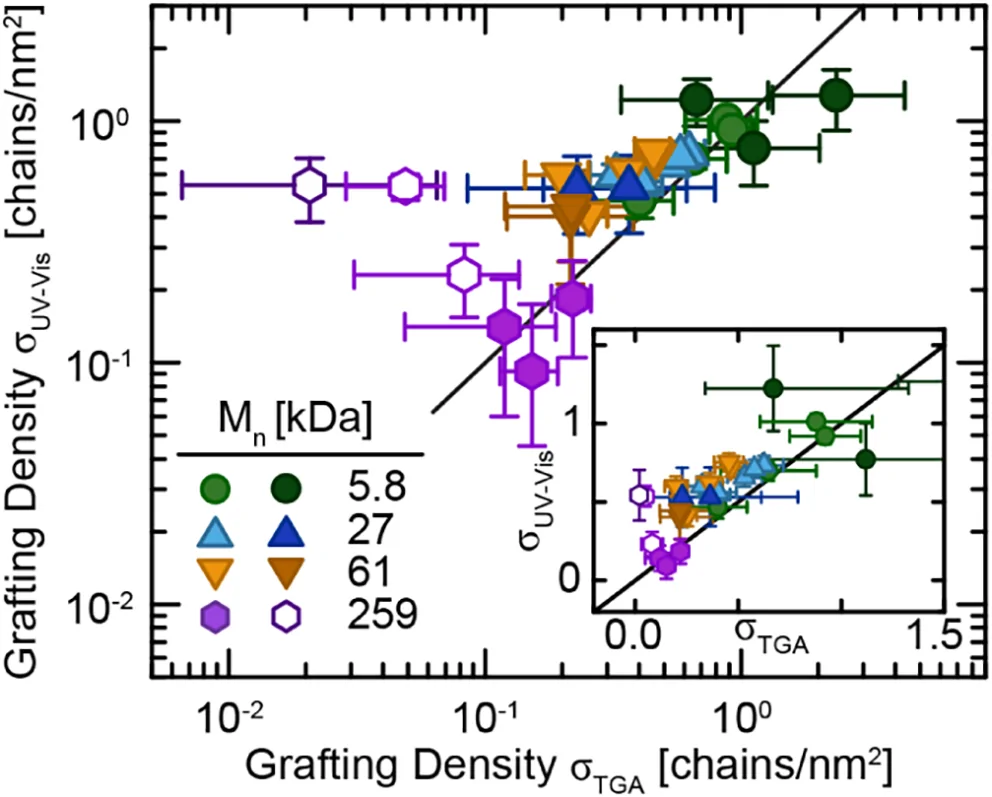
Comparison of σ obtained from the UV–vis method and traditional TGA. Light- and dark-colored symbols represent PGNPs with *R*_NP_ = 7 and 31 nm, respectively. The solid line *y* = *x* represents perfect quantitative agreement between the two methods. Open symbols represent experimental conditions that do not satisfy the assumptions of our model. *Inset:* The same comparison of σ obtained through both methods, plotted on a linear scale.

While most of the UV–vis measurements are consistent with TGA results, the open symbols in Figure 6 deviate noticeably from this trend and correspond to functionalizations performed with low concentrations of high *M*_n_ = 259 kDa PS-SH on both core sizes. Specifically, we find that the UV–vis approach significantly overestimates σ for these samples so that the data points sit well above the measured values from TGA. To understand this deviation, we investigate the role of PGNP length scales underlying the assumptions of our model (Figure 7). The optical sensitivity of PGNPs is governed by the decay length *L*, and our model assumes that the polymer maintains a cylindrical structure over this length scale. If the crossover radius *R*_c_ between SDPB and CPB is smaller than *R*_NP_ + *L*, however, the cylindrical chain approximation no longer holds because the chains adopt an SDPB structure near the particle surface.^19,75^ With this more coiled structure, the polymer chain will pass through a differential shell at a distance *z* from the particle surface multiple times, thereby contributing multiplicatively to the local dielectric properties. To explicitly identify this limit to our analytical approximation, we construct a state diagram using the TGA measurements of σ (Figure 7). We find that the deviating data points lie in the region in which (*R*_c_ – *R*_NP_)/*L* < 1, indicating that for these PGNPs, the SDPB transition occurs within the electromagnetic field decay length *L*. This state diagram therefore confirms the limits of our analytical approach in determining low grafting densities where the grafted polymer structure can no longer be reasonably described through the cylindrical approximation. Additionally, the state diagram identifies a second region in which the grafted layer thickness *h* is smaller than *L*. In this region, the pure solvent mixture beyond the grafted layer would contribute significantly to the peak shift. For the samples within this region, the UV–vis method remains in good quantitative agreement with TGA because the piecewise description of the polymer density profile φ­(*z*) in eq 3 accurately captures the optical properties of the neat solvent beyond the polymer shell.

**Figure 7:**
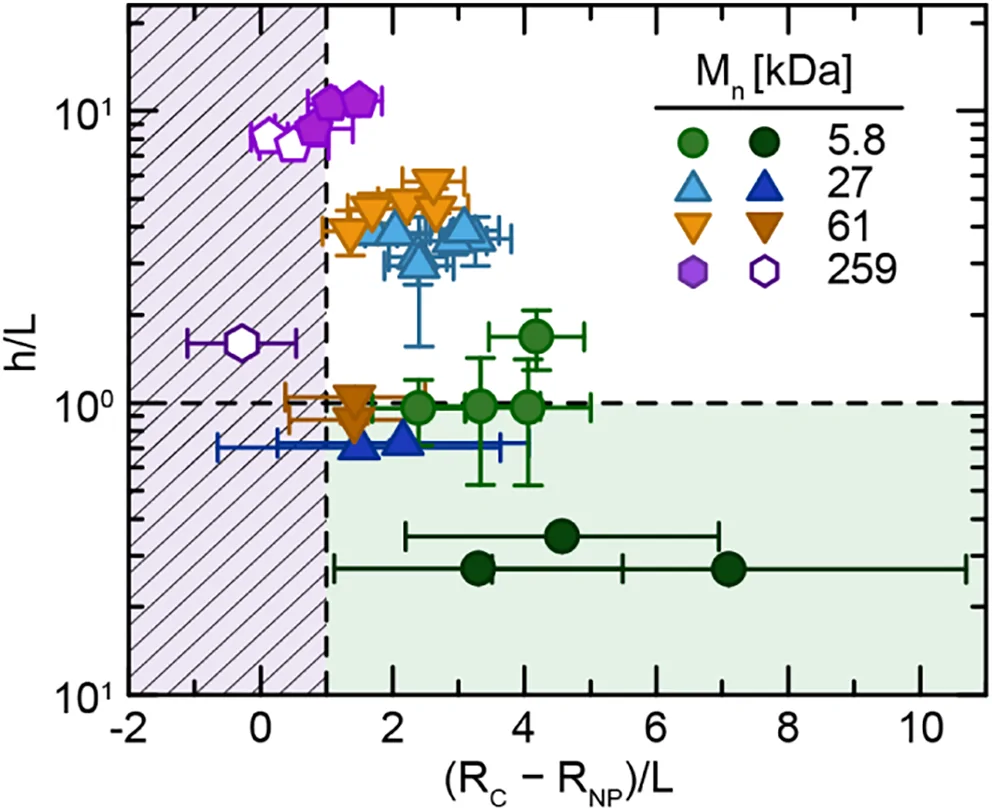
State diagram identifying regions in which UV–vis provides accurate estimates of σ. Light- and dark-colored symbols represent PGNPs with *R*_NP_ = 7 and 31 nm, respectively. Normalized polymer thickness *h*/*L* as a function of the normalized distance between the crossover radius and nanoparticle core size (*R*_c_ – *R*_NP_)/*L* for PGNPs with varying *M*_n_. The green shaded region indicates when the piecewise description of *n*_eff_ becomes important. The purple cross-hatched region indicates when the polymer can no longer be accurately approximated through a cylindrical assumption, resulting in overestimates of σ.

We end by noting that the choice of solvents used to vary *n* is critical for quantitative determination of σ. Because changes in solvent quality will change the conformation of the grafted polymer chains, it is imperative to maintain solvent quality while changing *n*. For solvent mixtures in which both are good solvents for the grafted polymer, such as THF–toluene and THF–anisole, we find nearly identical RIS values for PGNPs of both sizes. By contrast, when a mixture is formed by one good solvent and one θ-solvent, such as 2-butanone,^76,77^ we measure a significantly lower RIS (Supporting Information). We attribute this difference to conformational changes in the grafted polymer corona as we vary the solvent composition. DLS measurements of PGNPs in four different solvents support this interpretation, showing a smaller hydrodynamic size in 2-butanone than in the good solvents (Supporting Information). This reduction in hydrodynamic size in 2-butanone indicates that polymer chains are more contracted, invalidating the cylindrical polymer approximation, and therefore overestimating σ. These results highlight the importance of using solvent mixtures that maintain good-solvent conditions to minimize changes in polymer conformation during characterization.

## 3 Conclusion

In this work, we introduce a nondestructive method to measure polymer grafting density σ from the optical response of plasmonic nanoparticles. This method accurately quantifies σ across orders of magnitude in polymer *M*_n_, an order of magnitude in σ, and across multiple AuNP core sizes, in excellent agreement with standard TGA measurements. Our approach assumes that the polymer maintains an approximately cylindrical conformation across the electromagnetic decay length around the particle. If the polymer transitions into the SDPB regime and adopts a more coil-like conformation, our model overestimates σ. Additionally, our model requires good solvent quality across the studied refractive index range to achieve quantitatively accurate estimates. Despite these minor limitations, our optical method provides a new framework for determining σ on plasmonic nanoparticles, offering an affordable and straightforward alternative to conventional techniques. Notably, this method uses nondestructive technology widely available in most facilities that work with plasmonic nanoparticles. Unlike conventional techniques such as TGA, NMR, or ellipsometry, this approach does not require polymer cleavage to determine polymer *M*_n_, specialized instrumentation, or large sample quantities, and instead preserves the sample for further characterization and applications.

To facilitate reproducibility and broader use by the plasmonic nanoparticle community, the model and analysis code developed in this paper are made openly available.^78^ The model takes as input the LSPR peak positions λ_max_ for PGNPs measured across solvents of known refractive index *n*_solv_, the polymer shell thickness *h*, and key physical parameters including nanoparticle radius *R*_NP_, decay length *L*, cross sectional polymer diameter *b*, and polymer refractive index *n*_poly_. From these inputs, the code constructs the spatial polymer density profile φ­(*z*), converts it to a refractive index profile *n*(*z*), and computes an effective refractive index *n*_eff_ using near-field weighting. The σ is obtained by iteratively matching the calculated RIS as a function of *n*_eff_ to the RIS of the nanoparticle core. This approach allows users to extract quantitative estimates of σ directly from routine UV–vis data, providing a rapid and nondestructive alternative to conventional methods.

## 4 Materials and Methods

### 4.1 Materials

Sodium citrate tribasic dihydrate (Na_3_C_6_H_5_O_7_·2H_2_O, 99%), gold­(III) chloride trihydrate (HAuCl_4_.3H_2_O, 99.9%), silver nitrate (AgNO_3_, 99%), tris­(hydroxymethyl)­aminomethane ((HOCH_2_)_3_CNH_2_, 99.9%), glycerol (99.5%), anisole (99%), and 2-butanone (99%) were purchased from Sigma-Aldrich. *N,N*-dimethylformamide (DMF, 99.8%), tetrahydrofuran (THF, 99.0%) and toluene (99.5%) were obtained from VWR, Inc. Thiol-terminated polystyrene (PS-SH) with number-average molecular weight *M*_n_ of 5.8 kDa (dispersity Đ = *M*_w_/*M*_n_ = 1.1), 27 kDa (Đ = 1.07), 61 kDa (Đ = 1.08), and 259 kDa (Đ = 1.11) was purchased from Polymer Source and denoted as PS5.8, PS27, PS61, and PS259, respectively. Ultrapure water (18.2 MΩ·cm) was obtained from a Millipore Direct-Q5 UV-R system.

### 4.2 Synthesis and Functionalization of Gold Nanoparticles (AuNPs)

AuNPs with an average radius of *R*_NP_= 7 ± 1 nm were synthesized using a modified Turkevich method^79,80^ according to our previously reported protocol.^42^ Briefly, an aqueous solution of gold­(III) chloride was reduced with sodium citrate while stirring at 95 °C, resulting in an aqueous suspension of stable AuNPs. AuNPs with an average radius of *R*_NP_= 31 ± 4 nm were synthesized using the Tris-assisted premixing method.^81^ Briefly, aqueous solutions of gold­(III) chloride, sodium citrate, and silver nitrate were premixed at room temperature using a citrate-to-gold molar ratio of 1.1. After 4 min of premixing, the solution was added to boiling water containing Tris under vigorous stirring. The final concentrations of gold­(III) chloride, citrate, silver ion, and Tris in the reaction mixture were 0.25 mM, 0.27 mM, 5.05 μM, and 4 mM, respectively. Representative TEM images and the corresponding extinction spectrum of these larger AuNPs are provided in the Supporting Information.

AuNPs of both core sizes were functionalized with PS-SH by transferring dropwise into a DMF solution with varying amounts of PS–SH. This suspension was allowed to react under strong stirring at room temperature for 24 h.^42^ The PS–SH concentration serves as a key parameter for tuning the σ at each *M*_n_. The functionalized PGNPs were purified twice by repeated centrifugation–redispersion cycles in THF to remove unreacted polymer chains.

### 4.3 Transmission Electron Microscopy (TEM)

PGNPs were diluted in THF, pipetted directly onto Formvar/carbon-coated 400-mesh copper grids (Electron Microscopy Sciences), and allowed to dry under ambient conditions. PGNP nanostructures were then analyzed using transmission electron microscopy (TEM, JEOL JEM-F200) at an accelerating voltage of 200 kV. The TEM images were segmented based on pixel intensity using ImageJ^82^ to identify individual particles and quantify particle size. Particle size *R*_NP_ is determined from the major Feret diameter of the segmented areas.

### 4.4 Thermal Gravimetric Analysis (TGA)

PGNPs suspended in THF (∼3.5 μL) were carefully pipetted onto a clean platinum pan and subsequently dried in a vacuum oven to facilitate solvent evaporation, resulting in approximately 1 mg of dried PGNPs. TGA was then performed using a TA Instruments TGA 55 under a nitrogen flow of 25 mL min^–1^. Samples were heated from ambient temperature to 600 °C at a rate of 5 °C min^–1^. The polymer σ was calculated according to

6 $$\sigma_{\mathrm{TGA}}=\frac{m_{\mathrm{polymer}}{\;\rho }_{\mathrm{NP}}R_{\mathrm{NP}}N_{A}}{m_{\mathrm{NP}}\;3M_{n}}$$

where *m*_polymer_ and *m*_NP_ are the polymer and AuNP core masses respectively, ρ_NP_ is the core mass density, and *N*_A_ is Avogadro’s number.^19,83^ The *m*_polymer_ was determined by the mass loss at 280 °C to eliminate any contributions from residual THF and thiol ligands. The *m*_NP_ was determined by the remaining mass at 600 °C.

### 4.5 UV–Visible (UV–Vis) Spectroscopy

UV–vis extinction spectra of PGNP suspensions at low concentrations (0.02–0.04 mg mL^–1^) were acquired using a Shimadzu UV-2600i spectrophotometer at a scanning rate of 225 nm min^–1^ over a range of 400 to 900 nm. All measurements were performed at a constant temperature of 25 °C in glass cuvettes with a 1 cm optical path length. The dielectric environment surrounding the particles was varied by preparing solvent mixtures with distinct refractive indices. For bare AuNPs, water–glycerol mixtures were used to span a refractive index range of 1.3342 to 1.4400. For PGNPs, mixtures of THF and toluene were used to achieve refractive indices ranging from 1.4116 to 1.5027. The refractive index of each mixture was calculated using the volume fractions and refractive indices of the pure solvents based on the Lorentz–Lorenz equation,^69,84,85^ The LSPR peak wavelength λ_max_ was quantified from the extinction spectra by fitting the data to a Gaussian peak function.

### 4.6 Dynamic Light Scattering (DLS)

DLS analysis was performed on PGNP samples in good solvents (THF) to measure the hydrodynamic radius *R*_H_ at room temperature using a Brookhaven BI-200SM instrument with a wavelength of λ = 640 nm, and a 40 mW diode laser as the light source. All measurements were conducted at a 90^◦^ scattering angle to measure the scattered light intensity at 25 °C. The intensity autocorrelation function *G*_2_ was fit to a single stretched exponential function given by

7 $$G_{2}\left(q,\tau \right)=A+B\;\exp\;\left[-2{\left(\Gamma \tau \right)}^{\beta }\right]$$

where τ is the delay time, Γ is the relaxation rate, and β is a stretching exponent. *A* and *B* are constants that characterize the signal noise at long times and the signal amplitude at short times, respectively. The PGNP diffusivity was then obtained by *D* = Γ/*q* ^2^, where *q* = 4*π n* sin­(θ/2)/λ is the wavevector, and *n* is the index of refraction of THF. The corresponding hydrodynamic radius *R*_H_ was calculated by applying the Stokes–Einstein equation *R*_H_ = *k*_*B*_ *T*/6*π η D* where η is the temperature-dependent solvent viscosity with values of 0.46 mPa · s for THF and 0.89 mPa · s for water at 25 °C, *T* is the temperature, and *k*_*B*_ is the Boltzmann constant.

## Supplementary Material



## Notes

Details of the characterization of the large core PGNPs, the effect of different solvent qualities, experimental determination of the refractive-index sensitivity of AuNPs, the Mie-theory prediction of the refractive-index sensitivity of AuNPs, and the electromagnetic near-field decay-length calculation are provided (PDF)
