# Supplementary material for: A Nondestructive Method to Quantify Polymer Graft Density on Nanoparticles Using Optical Extinction Spectra
Source: ACS Nano. 2026 Sep 29;20(40):27274–83. doi: 10.1021/acsnano.6c11390 (PMC13671162; doi:10.1021/acsnano.6c11390)
Supplement: Supplementary file 1 [file nn6c11390_si_001.pdf]

Supporting Information: A non-destructive method to quantify polymer graft density on nanoparticles using optical extinction spectra

Masoud Abdi,† Irene Andreu,*,† and Ryan Poling-Skutvik*,†,‡

†*Department of Chemical, Biomolecular, and Materials Engineering, University of Rhode Island, Kingston, RI, 02881, USA*

‡*Department of Physics, University of Rhode Island, Kingston, RI, 02881, USA*

E-mail: iandreu@uri.edu; ryanps@uri.edu

Contents

TEM characterization and size distribution of AuNPs **S-3**

Characterization and optical analysis of PGNPs with large cores **S-4**

Experimental determination of the refractive index sensitivity of AuNPs **S-7**

Mie theory prediction of the refractive index sensitivity of AuNPs **S-10**

Decay length calculation **S-12**

Effect of solvent quality on grafting density determination **S-15**

Summary of Grafting Density Measurement Data **S-17**

TEM characterization and size distribution of AuNPs

TEM characterization confirms the successful synthesis of AuNPs with two distinct core sizes, with average radii of $R_{\mathrm{NP}} = 7 \pm 1$ nm and $R_{\mathrm{NP}} = 31 \pm 4$ nm (Fig. S1). The corresponding particle-size distributions obtained from TEM image analysis are shown in Figs. S2 and S3 for the AuNPs with $R_{\mathrm{NP}} = 7$ and 31, respectively.

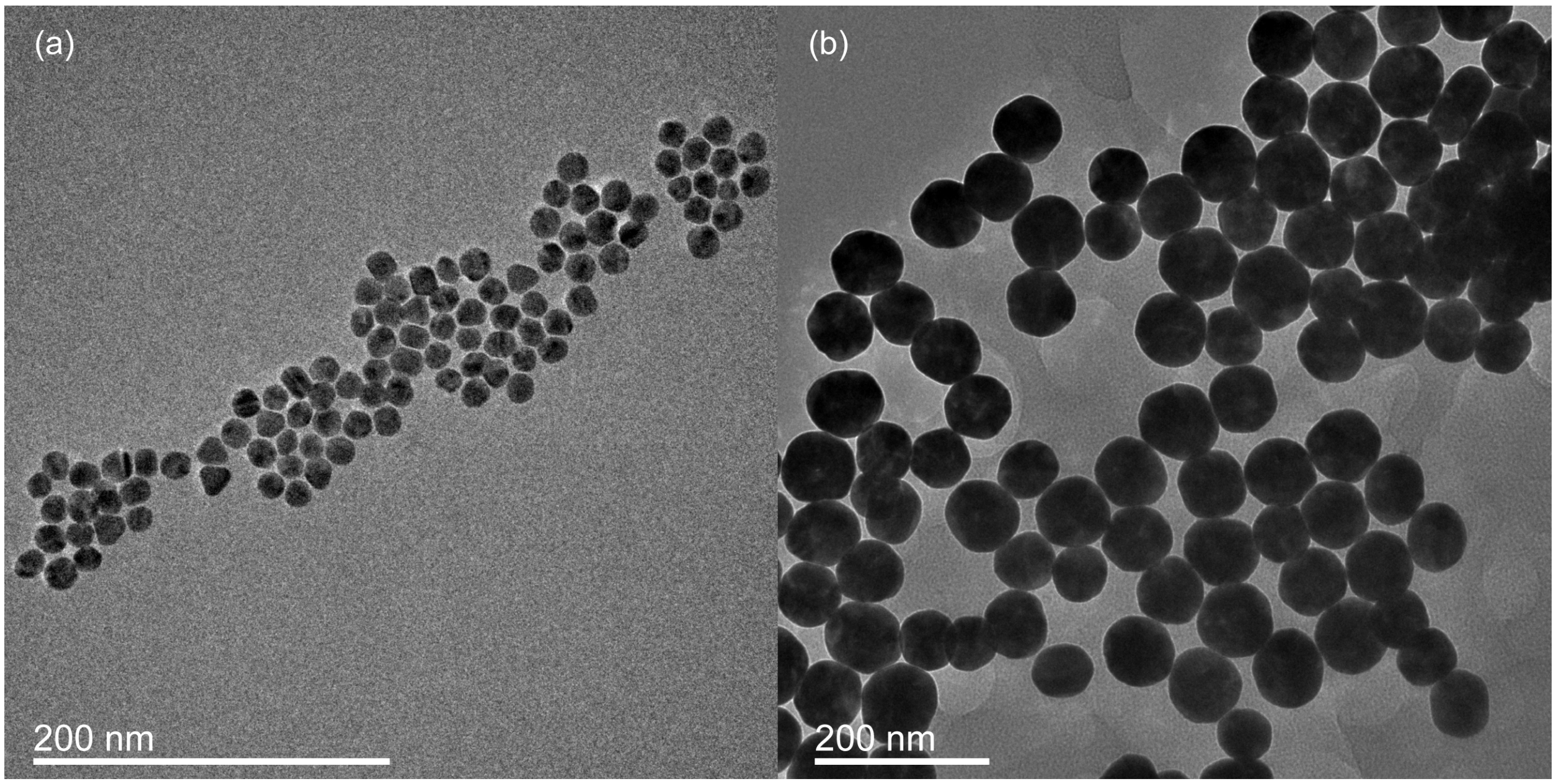

Figure S1: TEM images of synthesized AuNPs with average core radii of (a) $R_{\mathrm{NP}} = 7 \pm 1$ nm, (b) $R_{\mathrm{NP}} = 31 \pm 4$ nm.

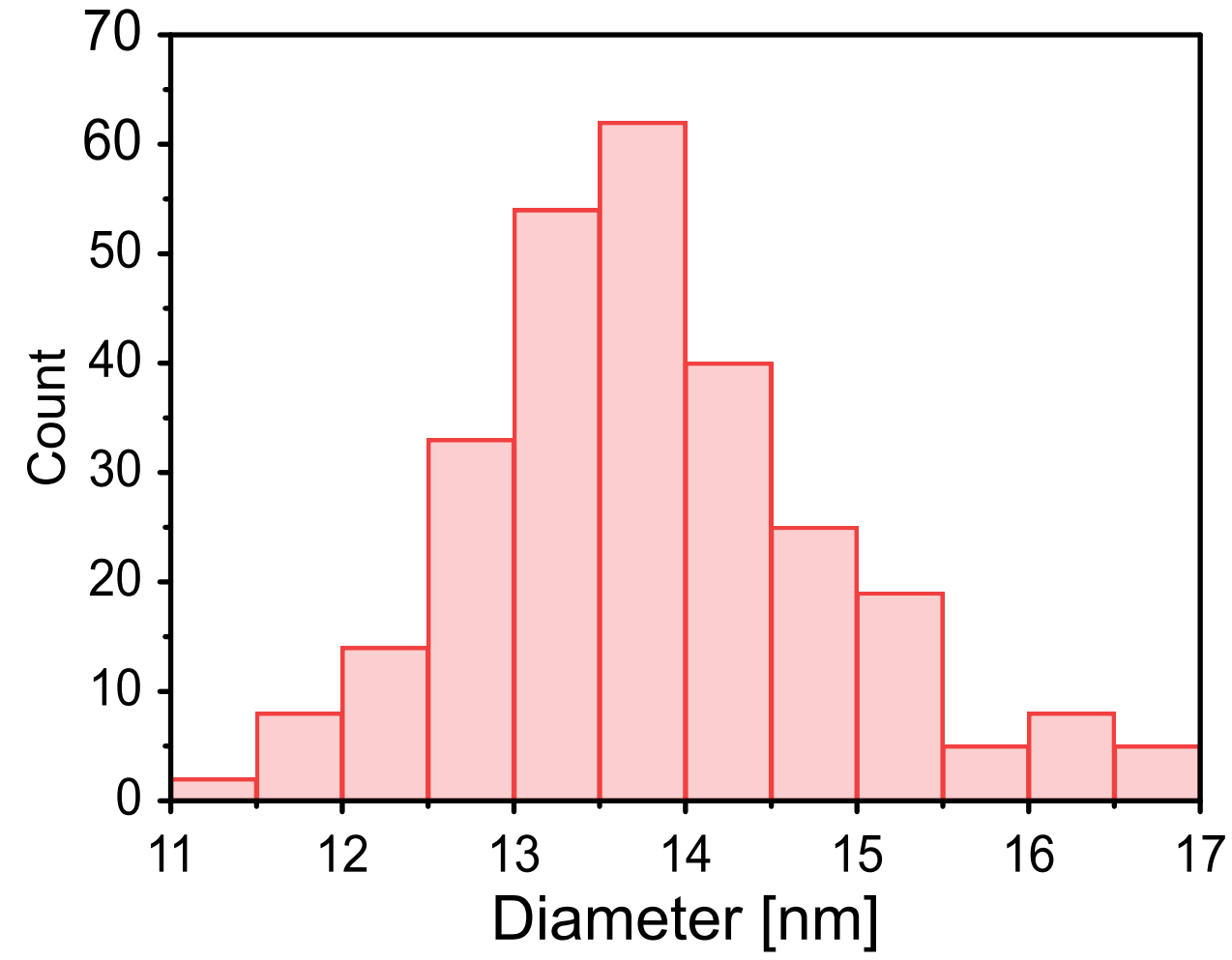

Figure S2: Size distribution of bare AuNPs with $R_{\mathrm{NP}} = 7$ nm obtained from TEM image analysis.

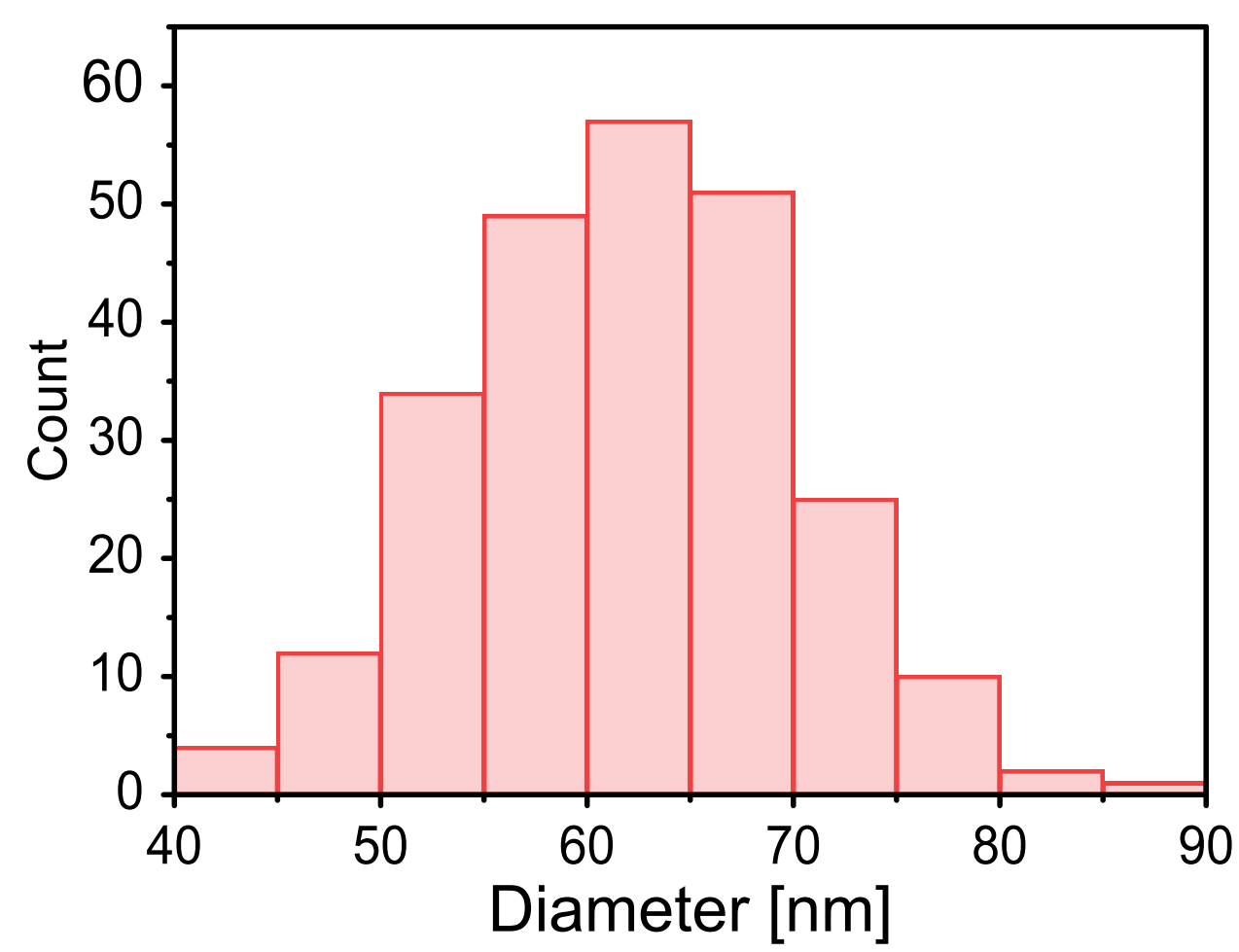

Figure S3: Size distribution of bare AuNPs with $R_{\mathrm{NP}} = 31$ nm obtained from TEM image analysis.

Characterization and optical analysis of PGNPs with large cores

TGA curves for PGNPs with $R_{\mathrm{NP}} = 31$ nm with different polymer M_{n} are shown in Fig. S4. These PGNPs exhibit significantly smaller mass loss than the PGNPs with $R_{\mathrm{NP}} = 7$ nm because the grafted polymer represents a smaller fraction of the total sample mass.

Therefore, the TGA signal is noisier and the calculated grafting density, σ_{TGA}, carries greater uncertainty.

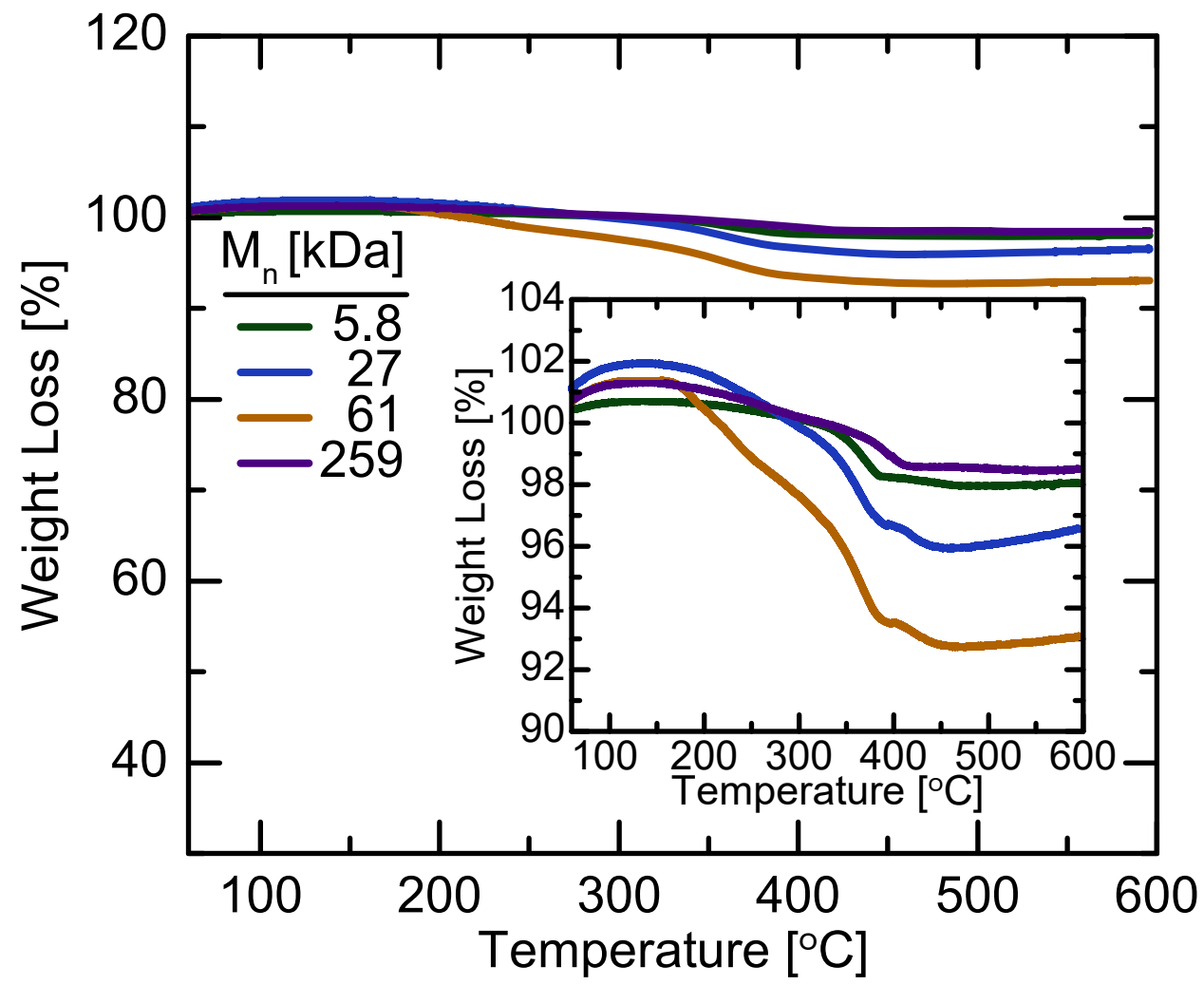

Figure S4: Weight loss of PGNPs with $R_{\mathrm{NP}} = 31$ nm functionalized with various M_{n} as a function of temperature under a heating rate of 5 °C min^{-1}.

All bare AuNPs and PGNPs exhibit narrow, symmetric LSPR peaks, as shown in Fig. S5, indicating that the particles remain stable and relatively uniform under these conditions. For all PGNPs, the LSPR peak is red-shifted relative to that of the bare AuNPs. This shift is expected because polystyrene has a higher refractive index than the surrounding solvent and therefore increases the local dielectric environment near the AuNP surface. In addition, PGNPs prepared with lower M_{n} exhibit larger red shifts, consistent with their higher σ.

The LSPR peak shifts extracted from the UV–vis spectra are plotted as a function of n_{solv} in Fig. S6. The PGNPs exhibit lower refractive index sensitivities than the bare AuNPs. To account for the contribution of the polymer corona to the local dielectric environment, n_{solv} is converted to an effective refractive index n_{eff}, as shown in Fig. S7. The low M_{n} PGNPs show larger n_{eff}, consistent with their higher grafting densities and greater polymer contribution within the plasmonic near field.

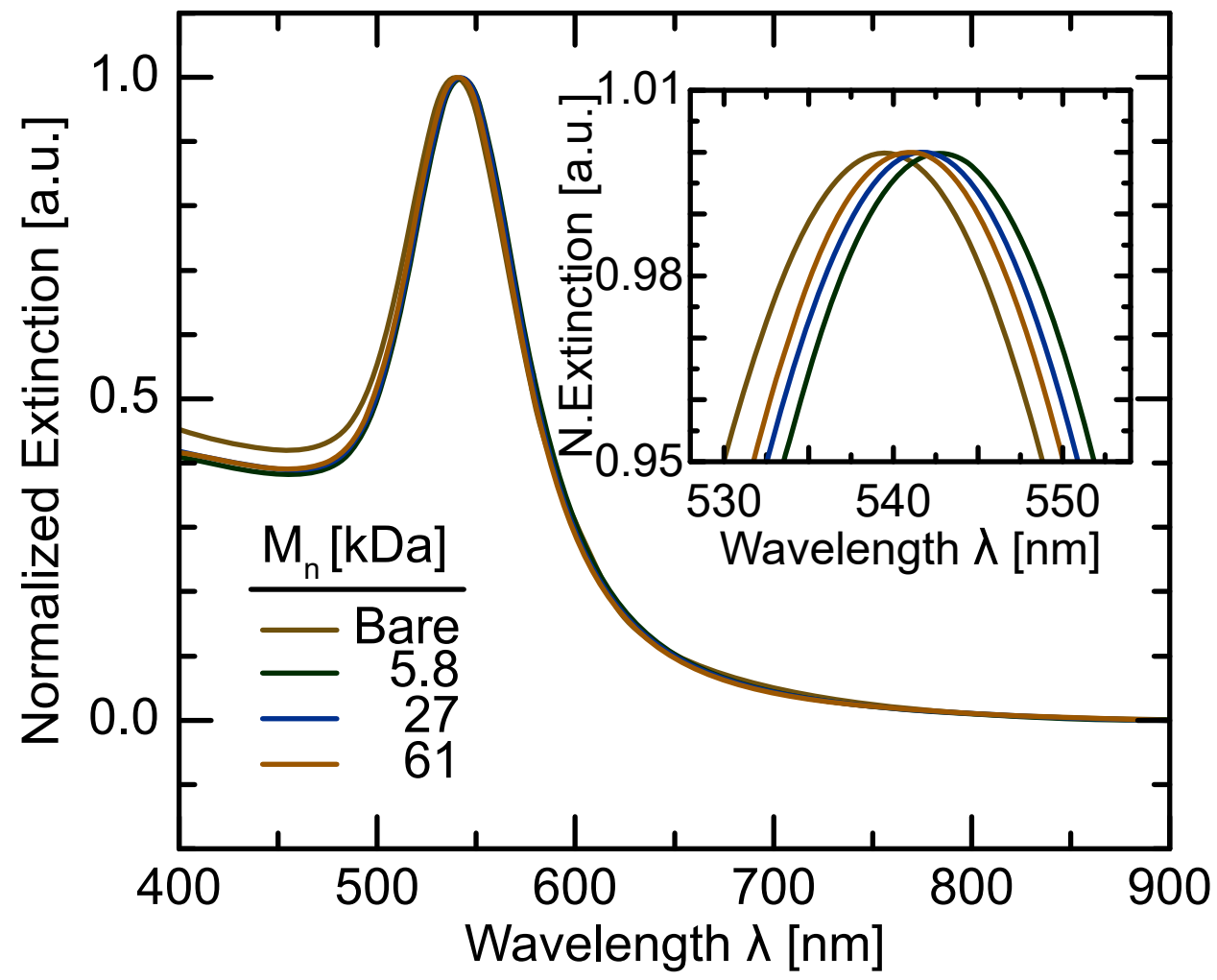

Figure S5: Normalized UV-vis extinction spectra for PGNPs with $R_{\text{NP}} = 31$ nm as a function of wavelength λ, for bare AuNPs in a water-glycerol solution ($n = 1.4116$) and PGNPs with different M_{n} in THF ($n = 1.4116$).

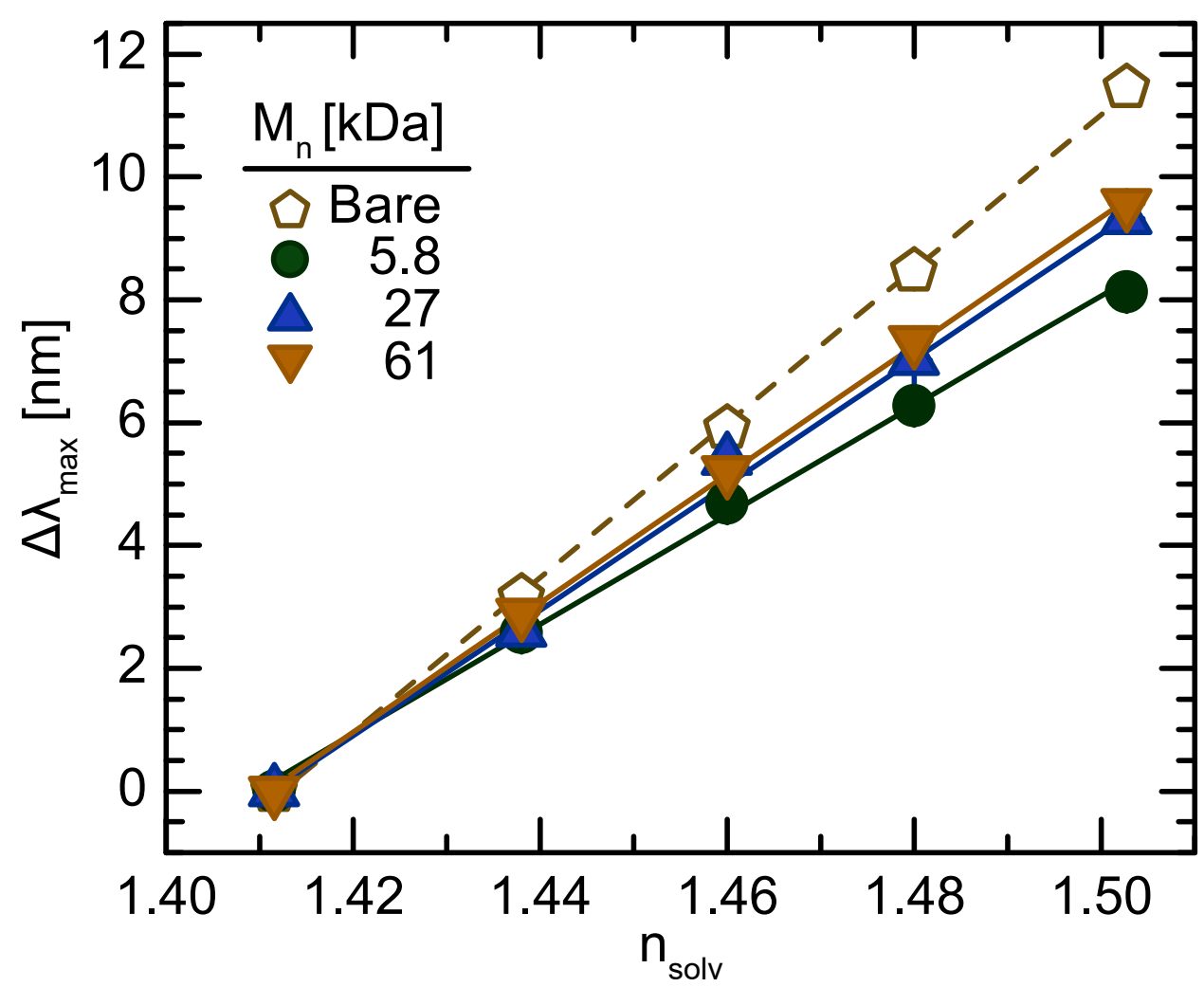

Figure S6: Peak shift $\Delta\lambda_{\text{max}}$ of PGNPs with $R_{\text{NP}} = 31$ nm (closed symbols) from pure THF and Mie theory predictions for bare AuNPs (open symbols) as a function of the refractive index of THF–toluene mixtures. Solid lines show linear fits to the experimental data, and dashed lines show linear fits to the calculated data.

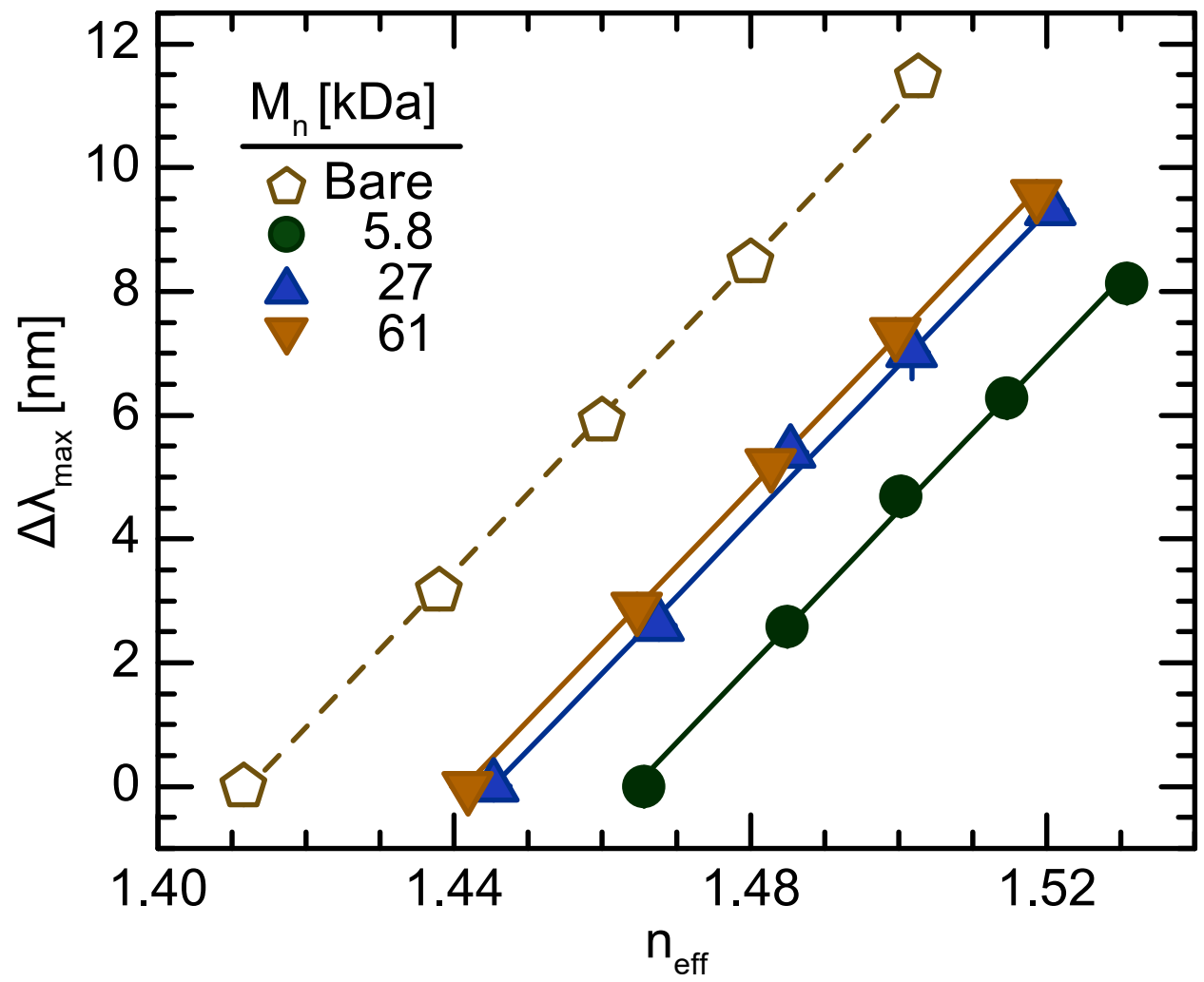

Figure S7: Mie theory predictions for bare AuNPs with $R_{\mathrm{NP}} = 31$ nm (open symbols) as a function of the refractive index of THF–toluene mixtures, and peak shift $\Delta\lambda_{\mathrm{max}}$ as a function of n_{eff} for PGNPs with $R_{\mathrm{NP}} = 31$ nm (closed symbols) with different M_{n}. The solid and dashed lines indicate linear fits showing a constant slope with $m = 125.5 \pm 1.5$.

Experimental determination of the refractive index sensitivity of AuNPs

Bare AuNPs with $R_{\mathrm{NP}} = 7$ and 31 nm are dispersed in a series of water–glycerol solutions spanning a range from $n = 1.3342$ to $n = 1.4400$. UV–vis extinction spectra of AuNPs with $R_{\mathrm{NP}} = 7$ nm are collected for each solution, as shown in Fig. S8. All samples show a symmetric localized surface plasmon resonance (LSPR) peak, indicating that the AuNPs are stable in these solutions. We observe red shifts with increasing n, and this red shift is expected because higher refractive indices increase the dielectric environment around the particles.

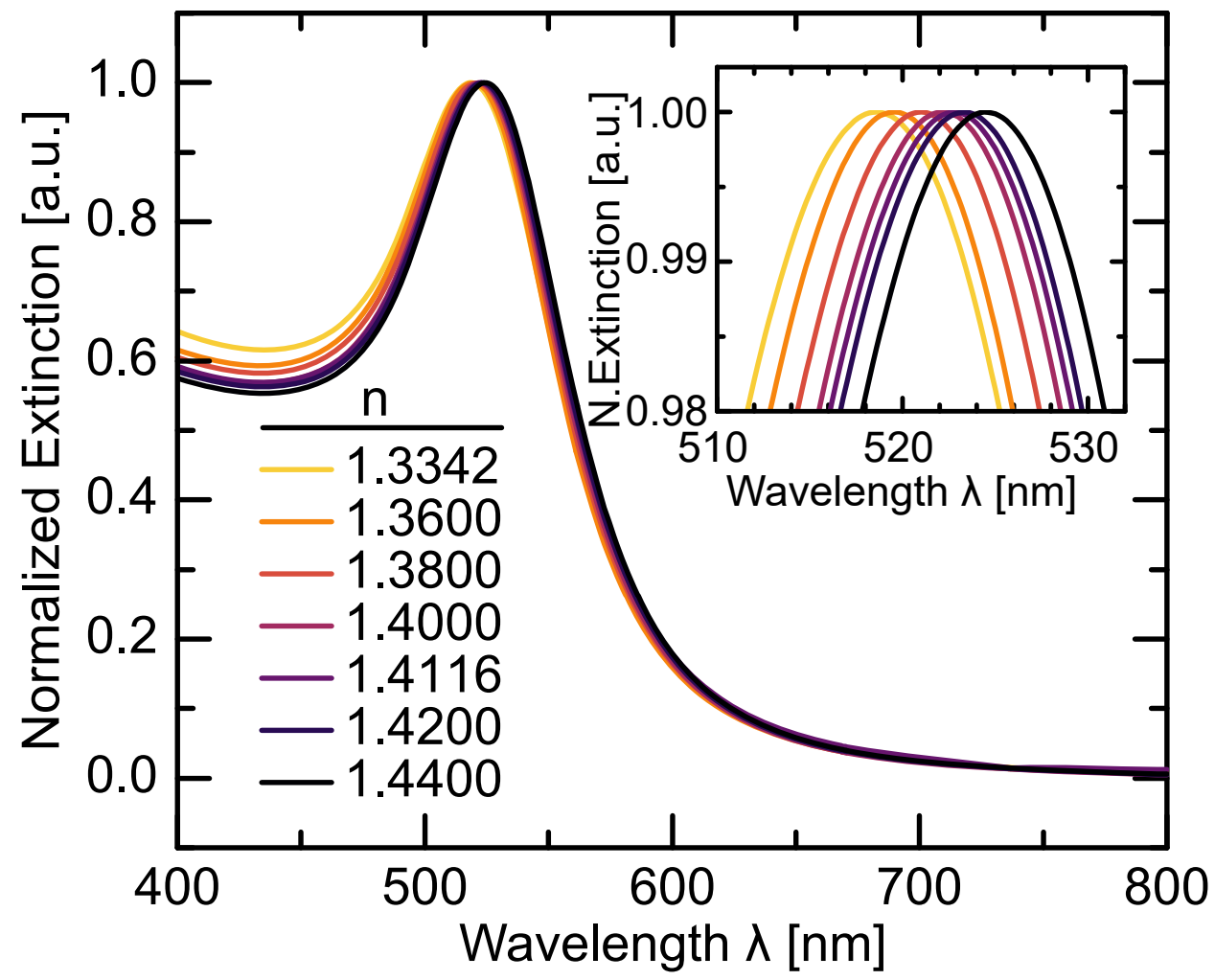

Figure S8: Normalized UV-Vis extinction spectra of bare AuNPs with $R_{\mathrm{NP}} = 7$ nm as a function of wavelength λ in water-glycerol solutions with varying refractive indices.

We plot the peak shift $\Delta\lambda_{\max}$ of samples from pure water $n = 1.3342$ as a function of the refractive index corresponding to the mixtures, as shown in Fig. S9, and S10. The measured $\Delta\lambda_{\max}$ varies linearly with n, consistent with earlier studies[S1–S3] and confirming that the LSPR peak shifts follow a linear dependence on the change in local refractive index according to

$$\Delta\lambda_{\max} = m\Delta n, \tag{S1}$$

where m is the refractive index sensitivity (RIS). Experimentally, the RIS is obtained from the slope of the linear fit $m = 58.9 \pm 1.7$ nm/RIU for AuNPs with $R_{\mathrm{NP}} = 7$ nm and $m = 117.5 \pm 0.9$ nm/RIU for AuNPs with $R_{\mathrm{NP}} = 31$ nm.

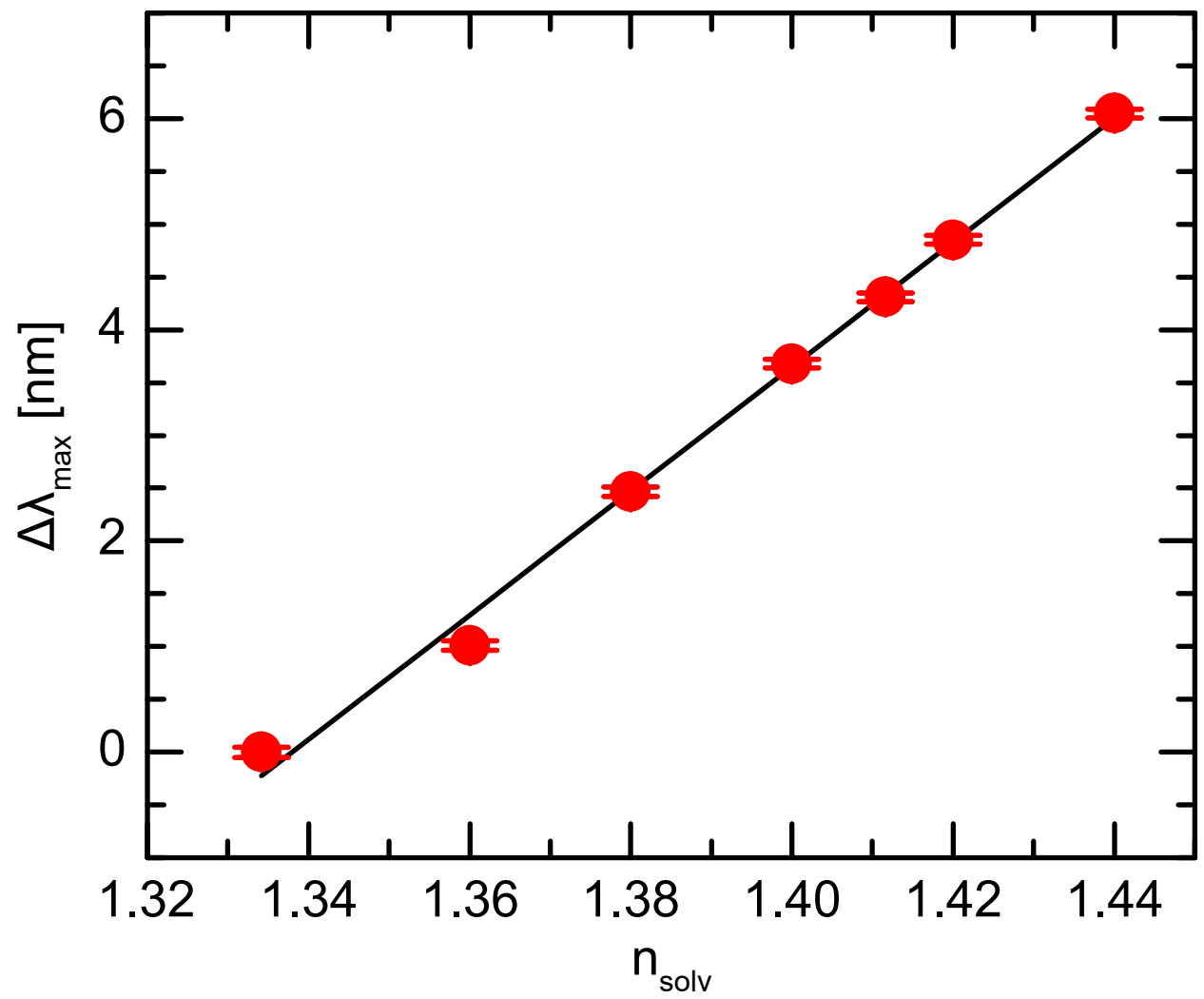

Figure S9: Peak shift $\Delta\lambda_{\rm max}$ of bare AuNPs with $R_{\rm NP} = 7$ nm from pure water as a function of the refractive index of water-glycerol mixtures. The solid line shows linear fits to the experimental data with a RIS of $m = 58.9 \pm 1.7$ nm/RIU.

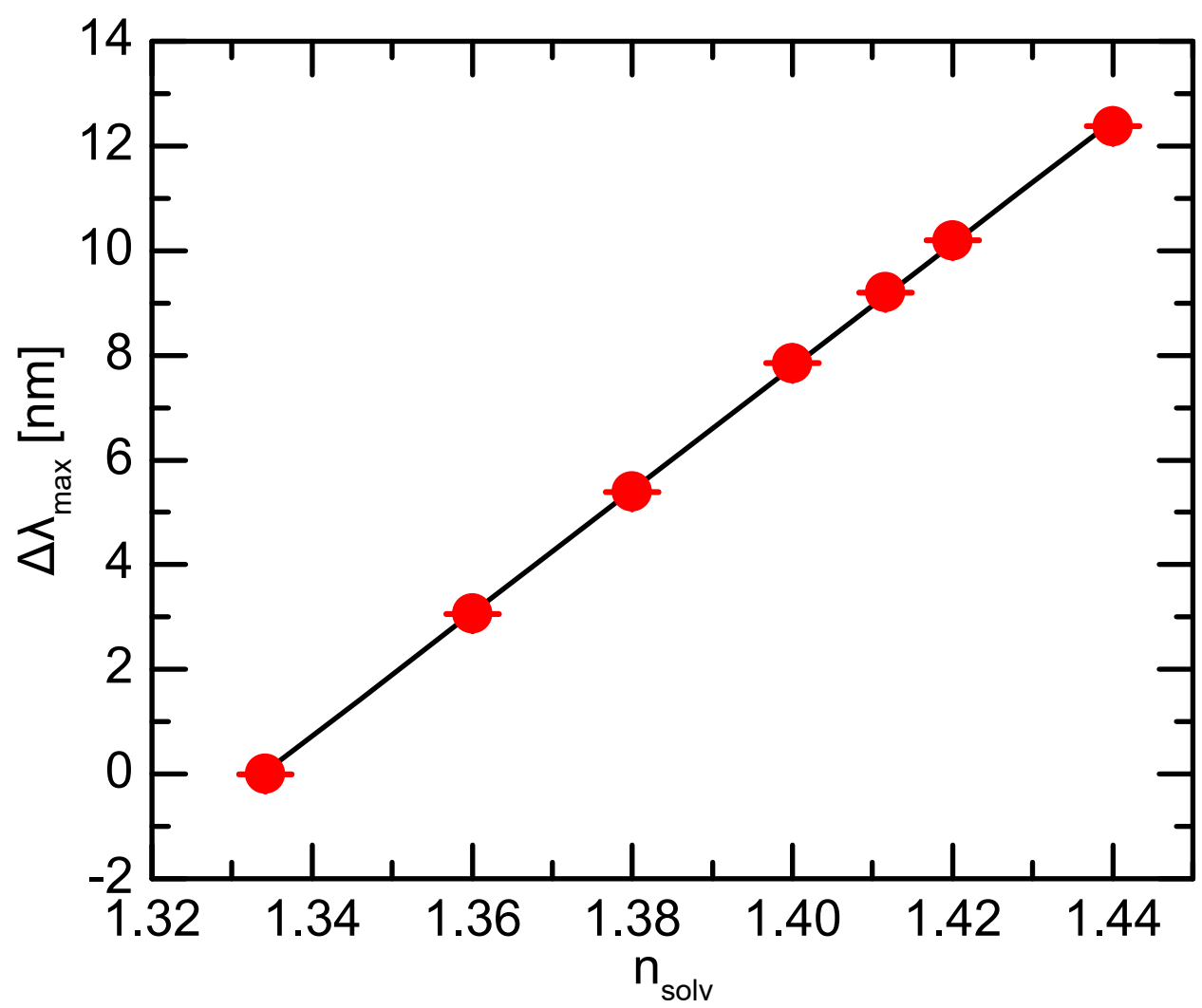

Figure S10: Peak shift $\Delta\lambda_{\rm max}$ of bare AuNPs with $R_{\rm NP} = 31$ nm from pure water as a function of the refractive index of water-glycerol mixtures. The solid line shows linear fits to the experimental data with a RIS of $m = 117.5 \pm 0.9$ nm/RIU.

Mie theory prediction of the refractive index sensitivity of AuNPs

We also predict the RIS of bare AuNPs for n in the range 1.3342 to 1.4400 using Mie theory. Here, we calculate the extinction spectra of bare AuNPs with $R_{\mathrm{NP}} = 7$ nm using the MiePlot software[S4,S5] with the McPeak dielectric function for gold,[S6] as shown in Fig. S11. These calculations exhibit excellent agreement with the measured LSPR peak of bare AuNPs suspended in water–glycerol solution. In Mie calculations, the peak shift $\Delta\lambda_{\mathrm{max}}$ for bare AuNPs with $R_{\mathrm{NP}} = 7$ nm and 31 nm from $n = 1.3342$ shows a linear change as a function of refractive index, as shown in Fig. S12 and Fig. S13, respectively. From Mie calculations, we find $m = 59.3 \pm 0.6$ and $m = 114.5 \pm 1.5$ nm/RIU for $R_{\mathrm{NP}} = 7$ nm and 31 nm, respectively, indicating that the RIS value in the Mie calculation agrees with the experimental value of AuNPs over the same range of n. This close agreement between Mie calculations and experimental data for water–glycerol solutions supports the use of Mie theory to estimate AuNP RIS values over other refractive index ranges relevant to the organic solvent and polymer corona environments. Over the range of n spanned by THF-toluene mixtures, Mie theory calculations for RIS of bare AuNPs with $R_{\mathrm{NP}} = 7$ nm yield $m = 64.5 \pm 0.6$, and for bare AuNPs with $R_{\mathrm{NP}} = 31$ nm an $m = 125.5 \pm 1.5$ as shown in Fig. 3(b) in the main text and Fig S14, respectively. We expect that the slight difference in m over different ranges of n results from the linear empirical approximation used to describe the dependence of the LSPR peak position on the surrounding refractive index.

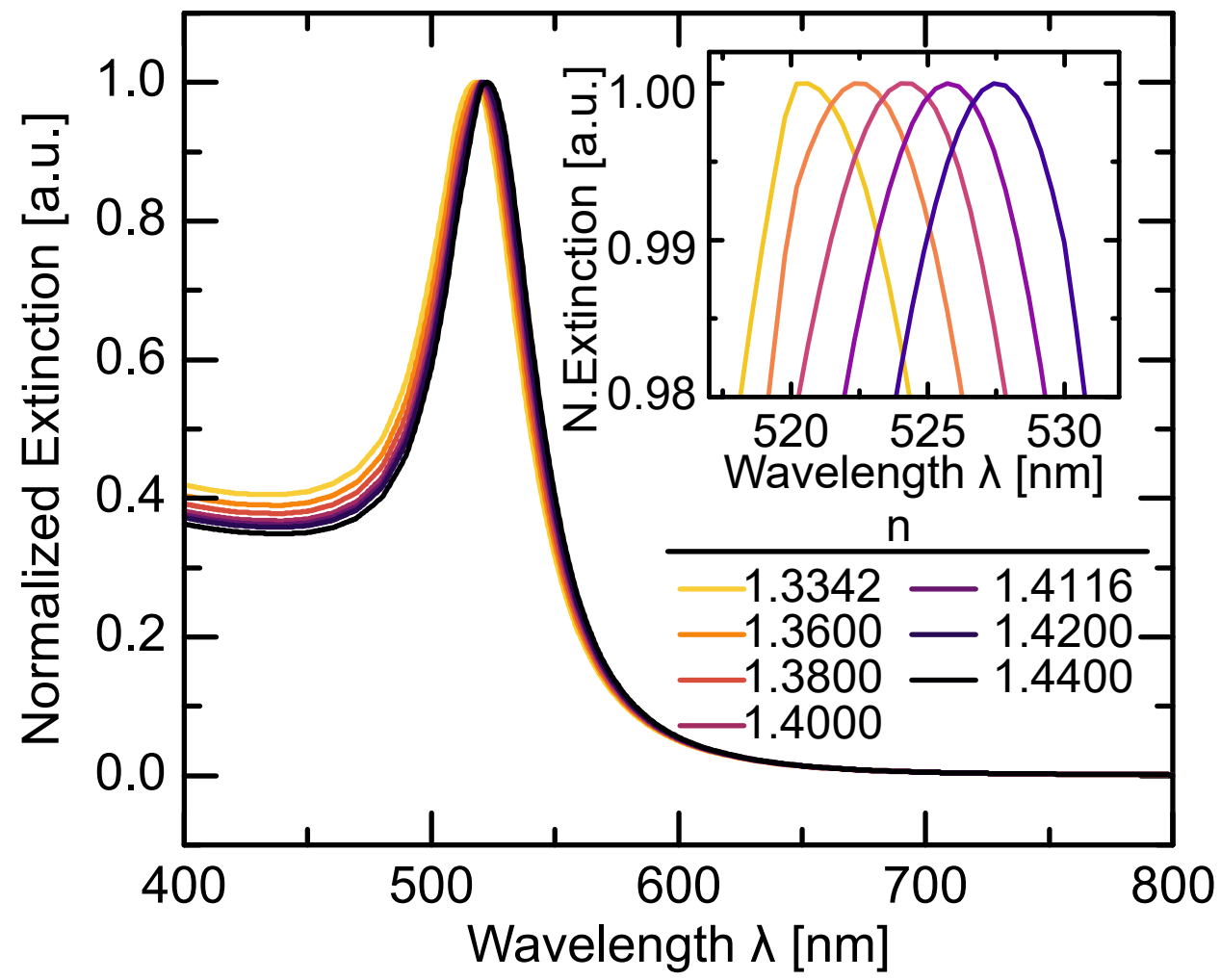

Figure S11: Predicted UV–vis extinction spectra as a function of wavelength λ from Mie theory for bare AuNPs with $R_{\mathrm{NP}} = 7$ nm in water-glycerol solutions with varying refractive indices.

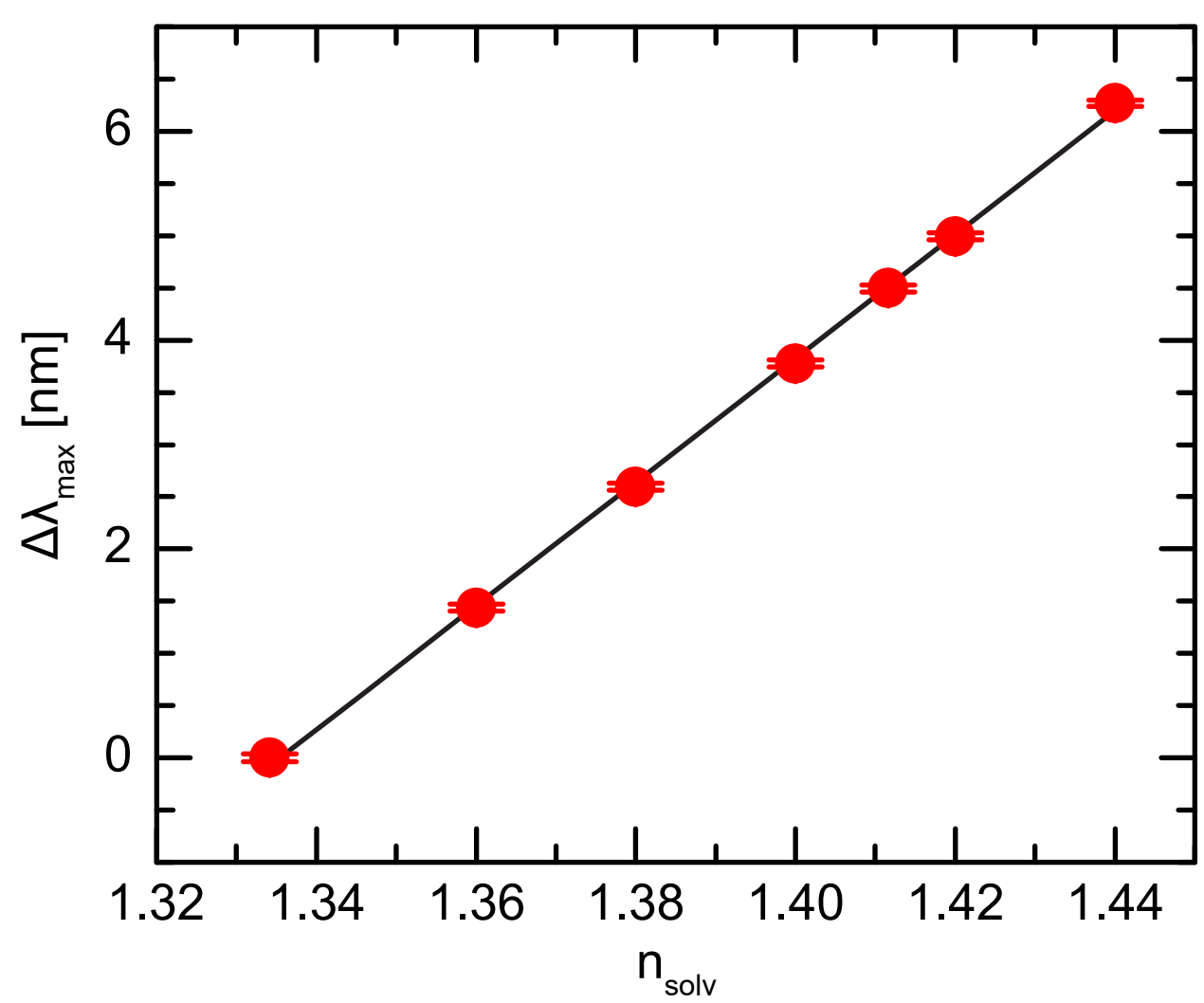

Figure S12: Mie theory predictions for bare AuNPs with $R_{\mathrm{NP}} = 7$ nm from pure water as a function of the refractive index of water-glycerol mixtures. The solid line shows linear fits to the calculated data, giving a RIS of $m = 59.3 \pm 0.6$ nm/RIU.

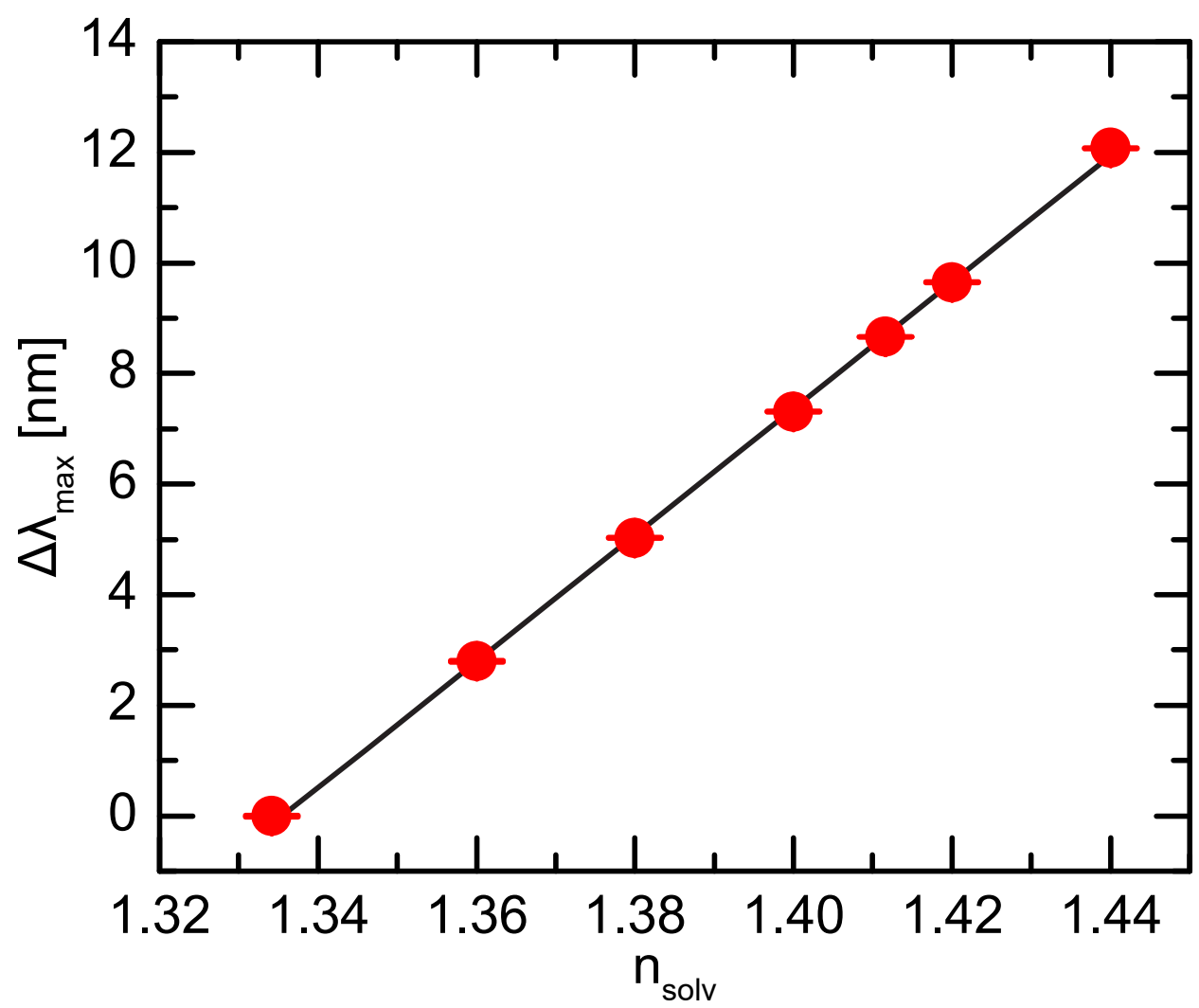

Figure S13: Mie theory predictions for bare AuNPs with $R_{\mathrm{NP}} = 31$ nm from pure water as a function of the refractive index of water-glycerol mixtures. The solid line shows linear fits to the calculated data, giving a RIS of $m = 114.5 \pm 1.5$ nm/RIU.

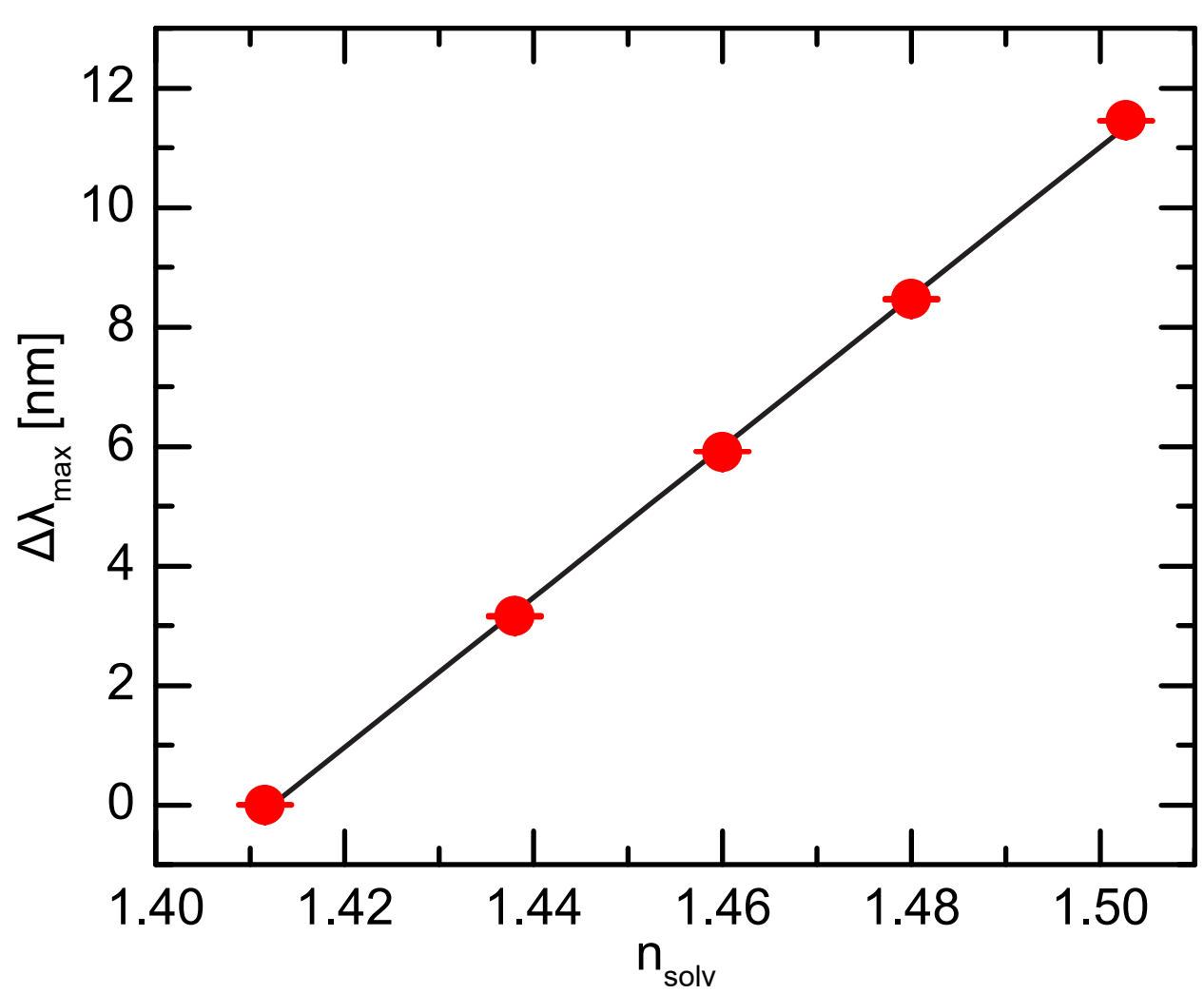

Figure S14: Mie theory predictions for bare AuNPs with $R_{\mathrm{NP}} = 31$ nm from pure water as a function of the refractive index of THF-toluene mixtures. The solid line shows linear fits to the calculated data, giving a RIS of $m = 125.5 \pm 1.5$ nm/RIU.

Decay length calculation

To determine the near-field interaction distance L, we calculate the LSPR response for AuNPs coated in polystyrene layers of varying thicknesses. The results of AuNPs with

$R_{NP} = 7$ nm are shown in Fig. S15 as an example. We observe red shifts with increasing thickness. These red shifts follow the expected exponential dependence given by

$$\Delta\lambda_{max} = m\Delta n \left[1 - \exp(-2d/L)\right], \tag{S2}$$

as shown in Fig. S16 and Fig. S17 for AuNPs with $R_{NP} = 7$ and 31 nm, respectively. The resulting fit finds the electromagnetic near-field decay length $L = 6.5 \pm 0.1$ and $L = 31.6 \pm 0.4$ nm for $R_{NP} = 7$ and 31 nm, respectively, which is in excellent agreement with literature values.[S1,S7]

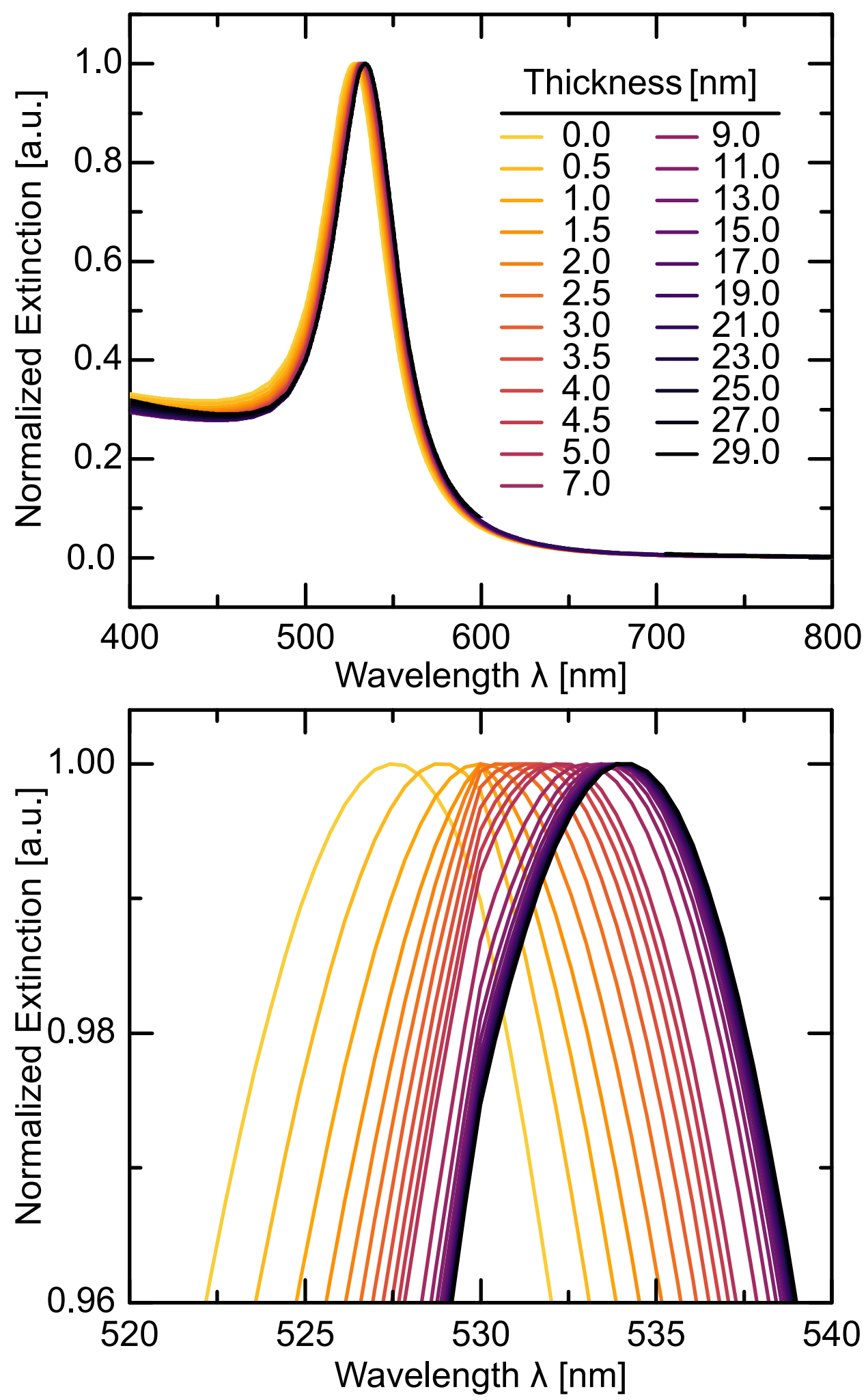

Figure S15: Mie theory predictions for the extinction spectra of AuNPs with $R_{NP} = 7$ nm coated with polystyrene shells of varying thicknesses from $d = 0.5$ to 29 nm in toluene.

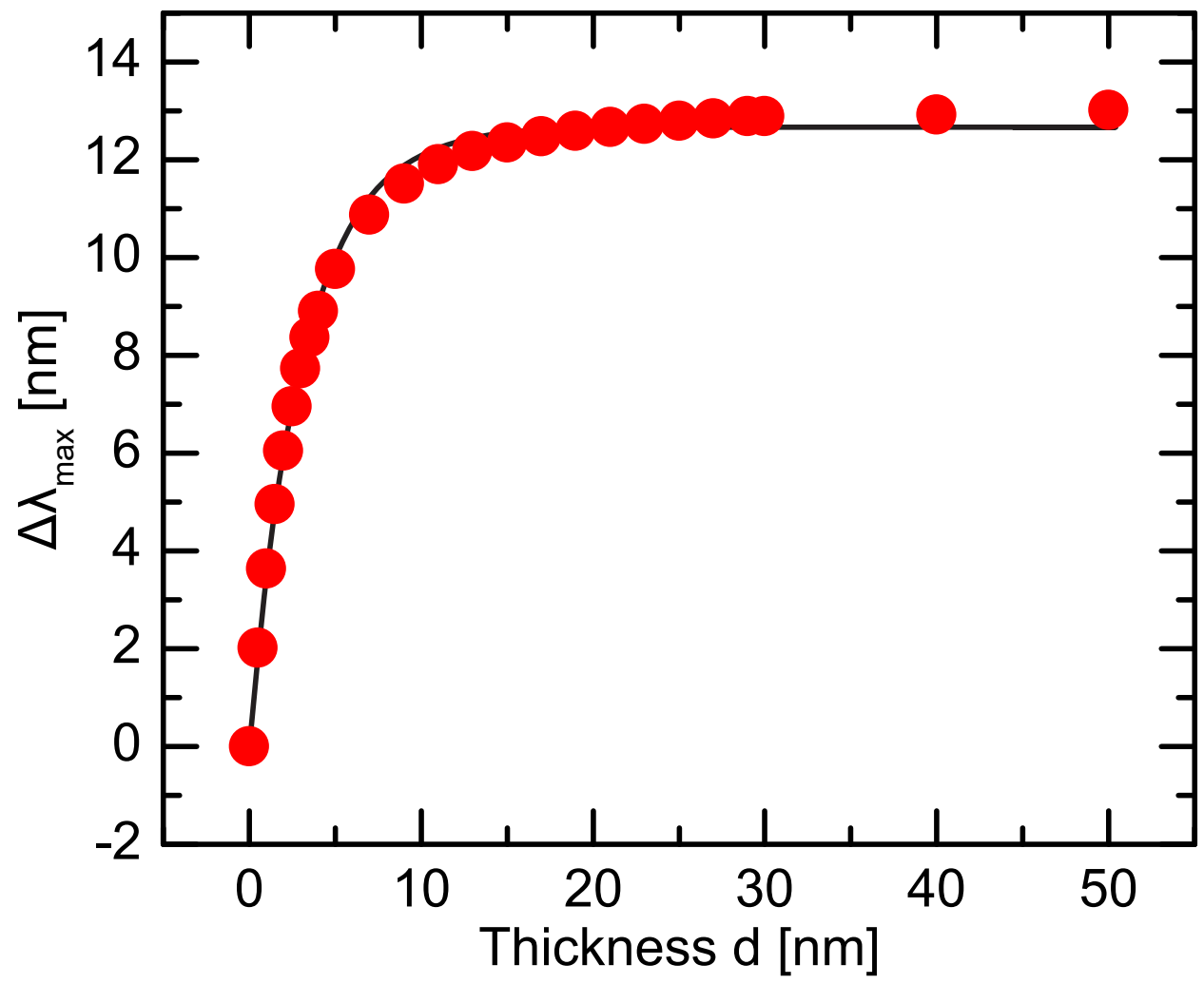

Figure S16: Peak shifts $\Delta\lambda_{\mathrm{max}}$ from Mie theory predictions as a function of polystyrene shell thickness d for AuNPs with $R_{\mathrm{NP}} = 7$ nm dispersed in THF. The solid line shows an exponential near-field fit with $L = 6.5 \pm 0.1$ nm.

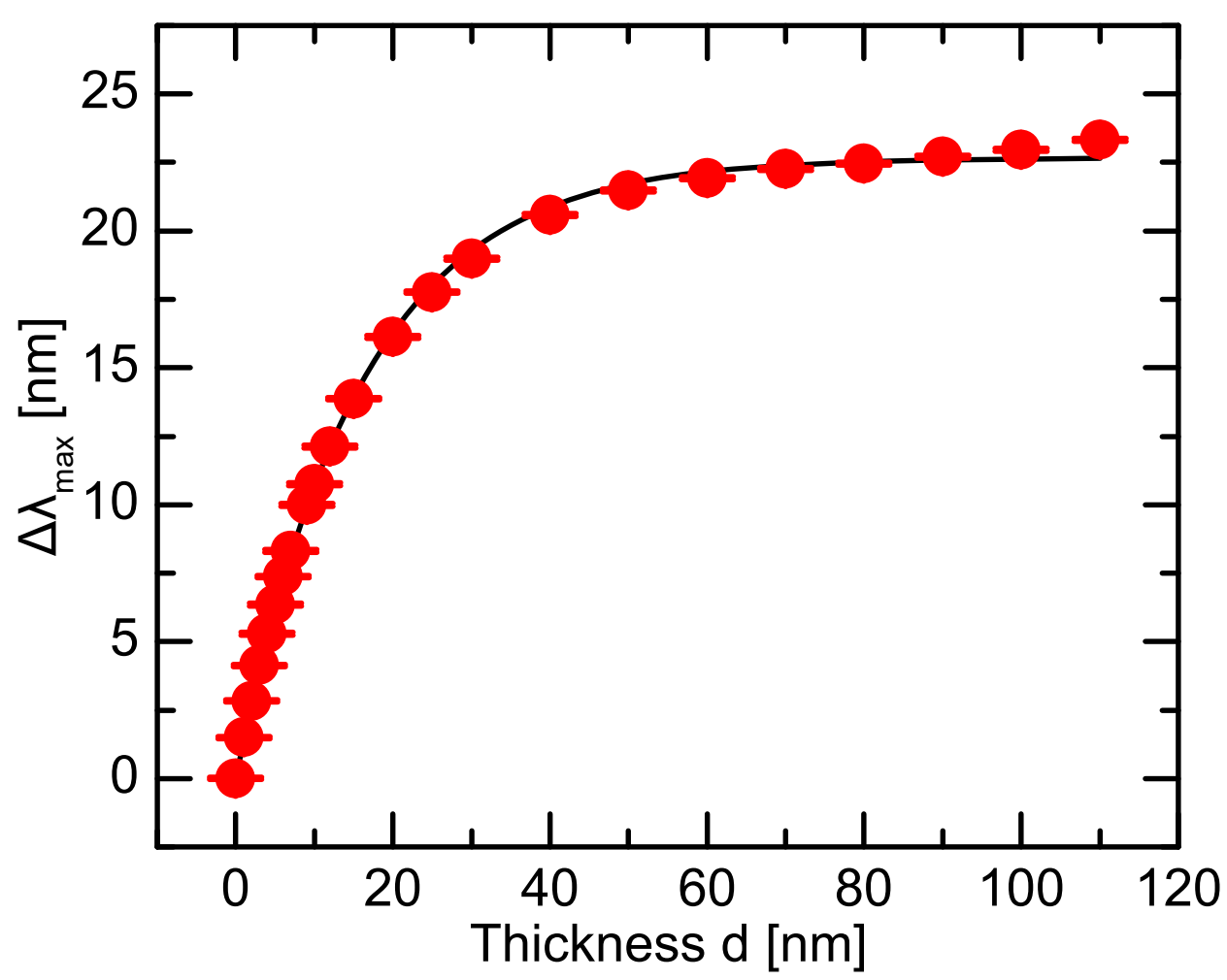

Figure S17: Peak shifts $\Delta\lambda_{\mathrm{max}}$ from Mie theory predictions as a function of polystyrene shell thickness d for AuNPs with $R_{\mathrm{NP}} = 31$ nm dispersed in THF. The solid line shows an exponential near-field fit with $L = 31.6 \pm 0.4$ nm.

Effect of solvent quality on grafting density determination

To evaluate the effect of solvent quality on the optical response of PGNPs, we measure the LSPR peak shift as a function of solvent refractive index using THF–toluene, THF–anisole, and 2-butanone–anisole mixtures. For both $R_{\mathrm{NP}} = 7$ nm and 31 nm PGNPs, the two good-solvent mixtures, THF–toluene and THF–anisole, give the same RIS, whereas the 2-butanone–anisole mixture exhibits a lower RIS (Fig. S18). This difference is consistent with changes in polymer conformation caused by the worse solvent quality of 2-butanone. DLS measurements in THF, toluene, anisole, and 2-butanone further support this interpretation. The autocorrelation functions in 2-butanone indicate a smaller hydrodynamic size relative to the good solvents (Fig. S19). These results show that solvent quality can change the polymer volume-fraction profile within the plasmonic near field and therefore affect the grafting density determined from the optical measurements.

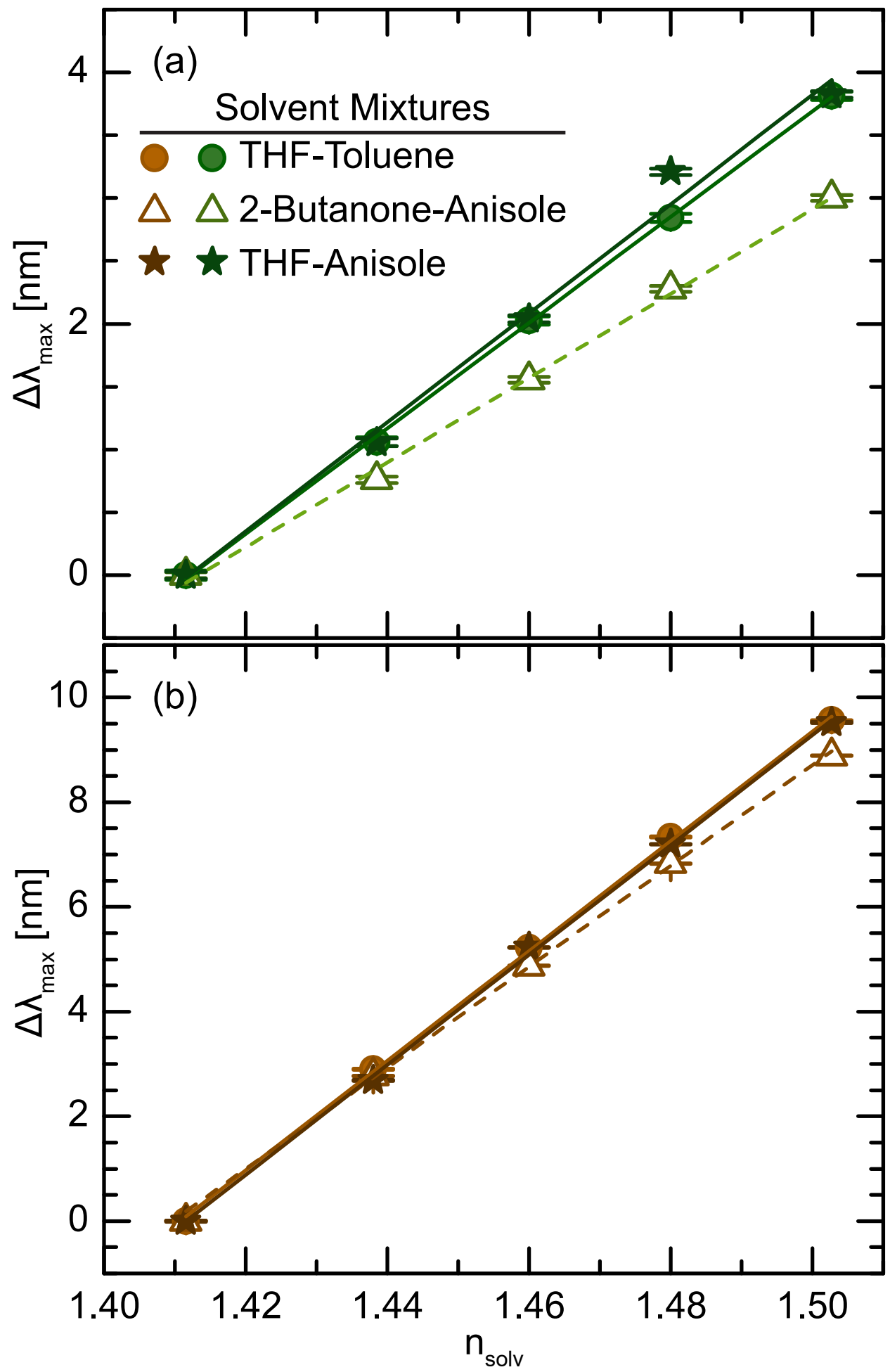

Figure S18: (a) LSPR peak shift $\Delta\lambda_{\text{max}}$ of AuNPs with $R_{\text{NP}} = 7$ nm functionalized with 5.8 kDa polystyrene as a function of solvent-mixture refractive index. (b) Corresponding results for AuNPs with $R_{\text{NP}} = 31$ nm functionalized with 61 kDa polystyrene. Solid lines represent linear fits for good-solvent mixtures, while dashed lines represent linear fits for the moderate-solvent mixture.

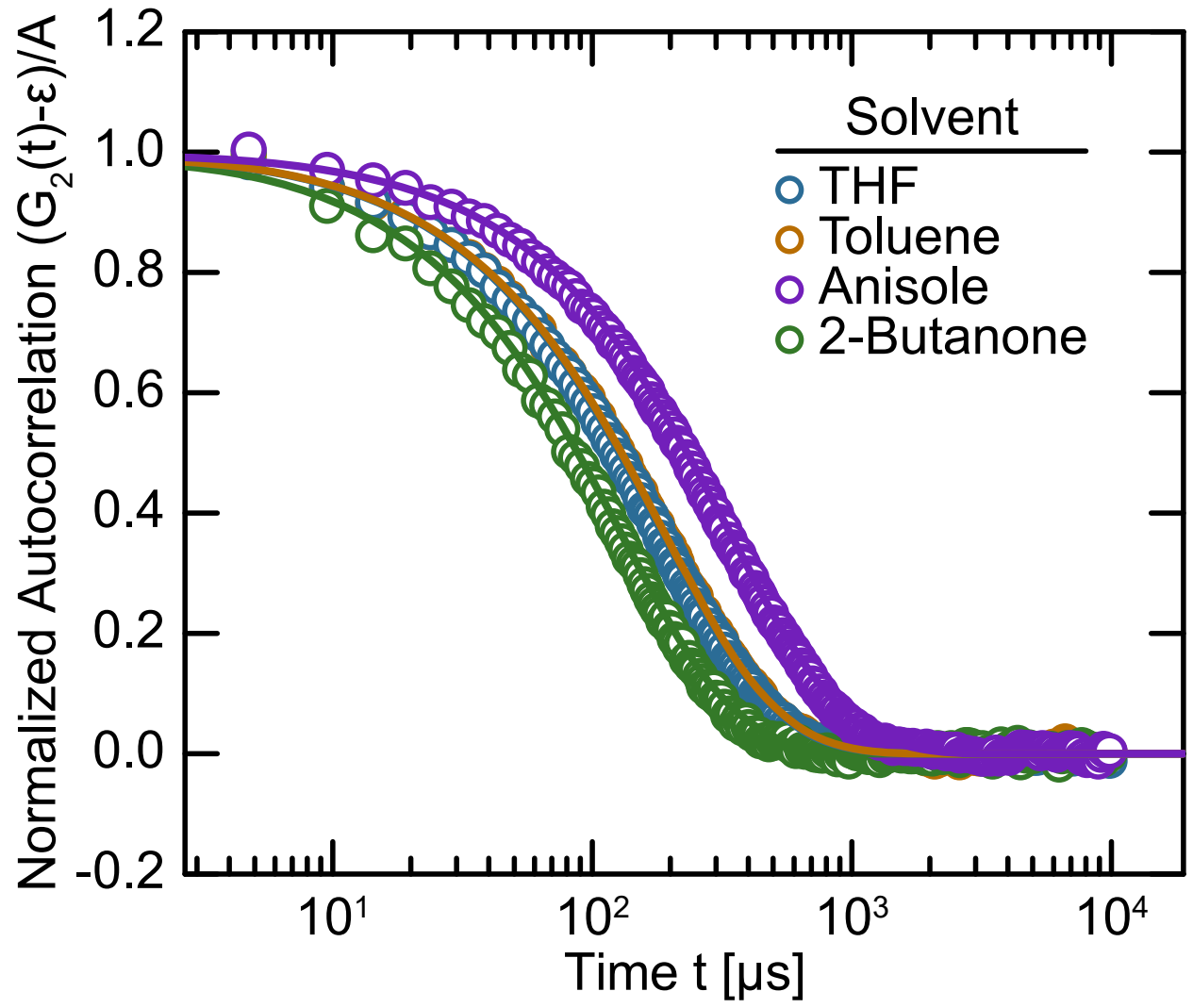

Figure S19: Normalized intensity autocorrelation functions $G_2(q, \tau)$ for AuNPs with $R_{\mathrm{NP}} =$ 31 nm functionalized with 61 kDa polystyrene at $q = 19.8\ \mu\mathrm{m}^{-1}$.

Summary of Grafting Density Measurement Data

The nominal M_{n} reported by the supplier, along with the M_{n} measured by gel permeation chromatography (GPC) and the corresponding dispersities of the polystyrene samples, are summarized in Table S1. The target grafting density is calculated from the stoichiometric ratio of polymer chains to AuNPs used during functionalization, normalized by the total AuNP surface area. The target values, together with the grafting densities determined by TGA and UV–Vis spectroscopy, are summarized in Table S2.

Table S1: Nominal and measured molecular weights and dispersities of the polystyrene samples.

**Nominal M_{n} (kDa)**	**Nominal Dispersity (Đ)**	**Measured M_{n} (kDa)**	**Measured Dispersity (Đ)**
5.8	1.1	5.3	1.11
27	1.07	28.0	1.12
61	1.05	49.0	1.19
259	1.11	153.2	1.57

Table S2: Grafting densities determined by TGA and UV-Vis measurements for PGNPs with different core radii and polymer M_n. Superscripts correspond to error in the positive direction and subscripts correspond to error in the negative direction.

R_{NP} **(nm)**	M_n **(kDa)**	**Target** σ **(chains nm**$^{-2}$**)**	σ_{TGA} **(chains nm**$^{-2}$**)**	σ_{UV-Vis} **(chains nm**$^{-2}$**)**
7	5.8	0.6	$0.40^{+0.14}_{-0.14}$	$0.47^{+0.07}_{-0.07}$
		0.9	$0.65^{+0.23}_{-0.23}$	$0.70^{+0.06}_{-0.06}$
		3	$0.88^{+0.27}_{-0.27}$	$1.01^{+0.05}_{-0.05}$
		4	$0.92^{+0.17}_{-0.17}$	$0.92^{+0.05}_{-0.05}$
	25	0.4	$0.40^{+0.06}_{-0.06}$	$0.55^{+0.07}_{-0.0}$
		0.5	$0.33^{+0.07}_{-0.07}$	$0.60^{+0.06}_{-0.06}$
		1	$0.40^{+0.09}_{-0.09}$	$0.56^{+0.06}_{-0.06}$
		1.5	$0.63^{+0.09}_{-0.09}$	$0.74^{+0.06}_{-0.06}$
		2	$0.53^{+0.08}_{-0.08}$	$0.66^{+0.06}_{-0.06}$
		4	$0.58^{+0.09}_{-0.09}$	$0.71^{+0.06}_{-0.06}$
	61	0.2	$0.20^{+0.06}_{-0.06}$	$0.60^{+0.06}_{-0.06}$
		0.4	$0.26^{+0.05}_{-0.05}$	$0.41^{+0.07}_{-0.07}$
		0.5	$0.36^{+0.06}_{-0.06}$	$0.62^{+0.06}_{-0.06}$
		1.5	$0.47^{+0.08}_{-0.08}$	$0.72^{+0.06}_{-0.06}$
		3	$0.45^{+0.07}_{-0.07}$	$0.75^{+0.06}_{-0.06}$
	259	0.2	$0.05^{+0.02}_{-0.02}$	$0.54^{+0.07}_{-0.07}$
		0.5	$0.08^{+0.05}_{-0.05}$	$0.23^{+0.08}_{-0.08}$
		1.5	$0.12^{+0.07}_{-0.07}$	$0.14^{+0.08}_{-0.08}$
		1.8	$0.15^{+0.04}_{-0.04}$	$0.09^{+0.08}_{-0.05}$
		1.9	$0.22^{+0.04}_{-0.04}$	$0.18^{+0.08}_{-0.08}$
31	5.8	0.9	$1.12^{+0.91}_{-0.50}$	$0.77^{+0.23}_{-0.23}$
		3	$2.36^{+2.00}_{-1.08}$	$1.27^{+0.36}_{-0.36}$
		5	$0.67^{+0.66}_{-0.33}$	$1.22^{+0.27}_{-0.27}$
	25	0.4	$0.23^{+0.38}_{-0.14}$	$0.53^{+0.19}_{-0.19}$
		1.5	$0.36^{+0.43}_{-0.20}$	$0.53^{+0.19}_{-0.19}$
	61	0.3	$0.22^{+0.16}_{-0.09}$	$0.40^{+0.19}_{-0.19}$
		0.6	$0.22^{+0.18}_{-0.10}$	$0.44^{+0.18}_{-0.18}$
	259	0.2	$0.02^{+0.04}_{-0.01}$	$0.54^{+0.16}_{-0.16}$

References

(S1) Jain, P. K.; Huang, W.; El-Sayed, M. A. On the Universal Scaling Behavior of the Distance Decay of Plasmon Coupling in Metal Nanoparticle Pairs: A Plasmon Ruler Equation. *Nano Lett.* **2007**, *7*, 2080–2088.

(S2) Jung, L. S.; Campbell, C. T.; Chinowsky, T. M.; Mar, M. N.; Yee, S. S. Quantitative Interpretation of the Response of Surface Plasmon Resonance Sensors to Adsorbed Films. *Langmuir* **1998**, *14*, 5636–5648.

(S3) Bukasov, R.; Ali, T. A.; Nordlander, P.; Shumaker-Parry, J. S. Probing the Plasmonic Near-Field of Gold Nanocrescent Antennas. *ACS Nano* **2010**, *4*, 6639–6650.

(S4) Laven, P. Simulation of Rainbows, Coronas and Glories Using Mie Theory and the Debye Series. *J. Quant. Spectrosc. Radiat. Transf.* **2004**, *89*, 257–269.

(S5) Laven, P. MiePlot: A Computer Program for Scattering of Light from a Sphere Using Mie Theory. http://www.philiplaven.com/mieplot.htm.

(S6) McPeak, K. M.; Jayanti, S. V.; Kress, S. J. P.; Meyer, S.; Iotti, S.; Rossinelli, A.; Norris, D. J. Plasmonic Films Can Easily Be Better: Rules and Recipes. *ACS Photonics* **2015**, *2*, 326–333.

(S7) Dileseigres, A. S.; Prado, Y.; Pluchery, O. How to Use Localized Surface Plasmon for Monitoring the Adsorption of Thiol Molecules on Gold Nanoparticles? *Nanomaterials* **2022**, *12*, 292.
